# Selective Degradation of Polyurethanes in Mixed Plastic Wastes via Ir‐Catalyzed Hydrogenolysis

**DOI:** 10.1002/anie.4288189

**Published:** 2026-07-08

**Authors:** Yuto Yamada, Takanori Iwasaki, Shinji Tanaka, Kyoko Nozaki

**Affiliations:** ^1^ Department of Chemistry and Biotechnology Graduate School of Engineering The University of Tokyo Bunkyo‐ku Tokyo Japan; ^2^ Department of Applied Chemistry Graduate School of Engineering Kyushu University Nishi‐ku Fukuoka Japan; ^3^ Research Institute for Chemical Process Technology National Institute of Advanced Industrial Science and Technology (AIST) Ibaraki Japan

**Keywords:** chemical recycling, chemoselectivity, homogenous catalysis, hydrogenolysis, urethanes

## Abstract

Chemical recycling of plastic waste has been widely undertaken to address a circular carbon economy. However, the presence of different polymers or composites in plastic waste complicates its chemical degradation into reusable chemical feedstocks. Here, we report the selective degradation of polyurethanes in mixed plastics with polyesters and polyamides using H_2_ as a reactant, contrarily to the implicitly low reactivity of urethanes against esters and amides. This chemoselectivity enables selective chemical recycling of urethanes in mixed textiles and end‐of‐life car seats, while thermoplastic polyesters and polyamides can be separated for mechanical recycling. This innovative recycling approach facilitates composite separation and resource circulation in mixed plastic waste, addressing a pressing global concern.

## Graphical abstract

The selective degradation of polyurethanes in mixed plastic wastes with polyesters and polyamides was achieved using H_2_ as a reactant, enabling simultaneous recovery of diformamides and polyols arising from polyurethanes and separation of polyesters and polyamides without any degradation. This innovative recycling approach facilitates composite separation and resource circulation in mixed plastic waste, addressing a pressing global concern.

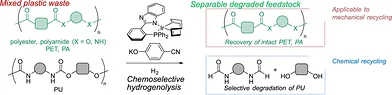

## 1 Introduction

The use of plastics has grown rapidly due to the versatility and cost‐effectiveness of polymer materials. Consequently, the amount of plastic waste generated globally has increased annually. According to recent projections from the OECD (Organisation for Economic Co‐operation and Development), the amount of plastic used is forecasted to increase nearly threefold, from 460 Mt in 2019 to 1,231 Mt in 2060 [1]. In pursuit of a sustainable society and a circular economy, efficient recycling of plastic waste has become an urgent global issue. Mechanical recycling, a key material‐recycling approach, remains the most used method for polymer recovery [2, 3]. The advantages of mechanical recycling include cost‐effectiveness, low energy consumption, and economic sustainability [4]. However, mechanical recycling has several limitations. It is generally applicable only to single‐polymer plastics and is poorly suited to mixed materials, such as polymer‐blended textiles and contaminated plastics [2]. Furthermore, it cannot be applied to thermosets, which account for 15%–20% of the global polymer production [5]. To overcome these obstacles and enable more comprehensive recycling of diverse plastic waste streams, it is essential to develop efficient and selective chemical recycling methods that enable the reproduction of virgin plastics via pure monomer recovery. This would complement the advantages of mechanical recycling and integrate the strengths of both approaches toward a more sustainable circular economy.

**FIGURE 1:**
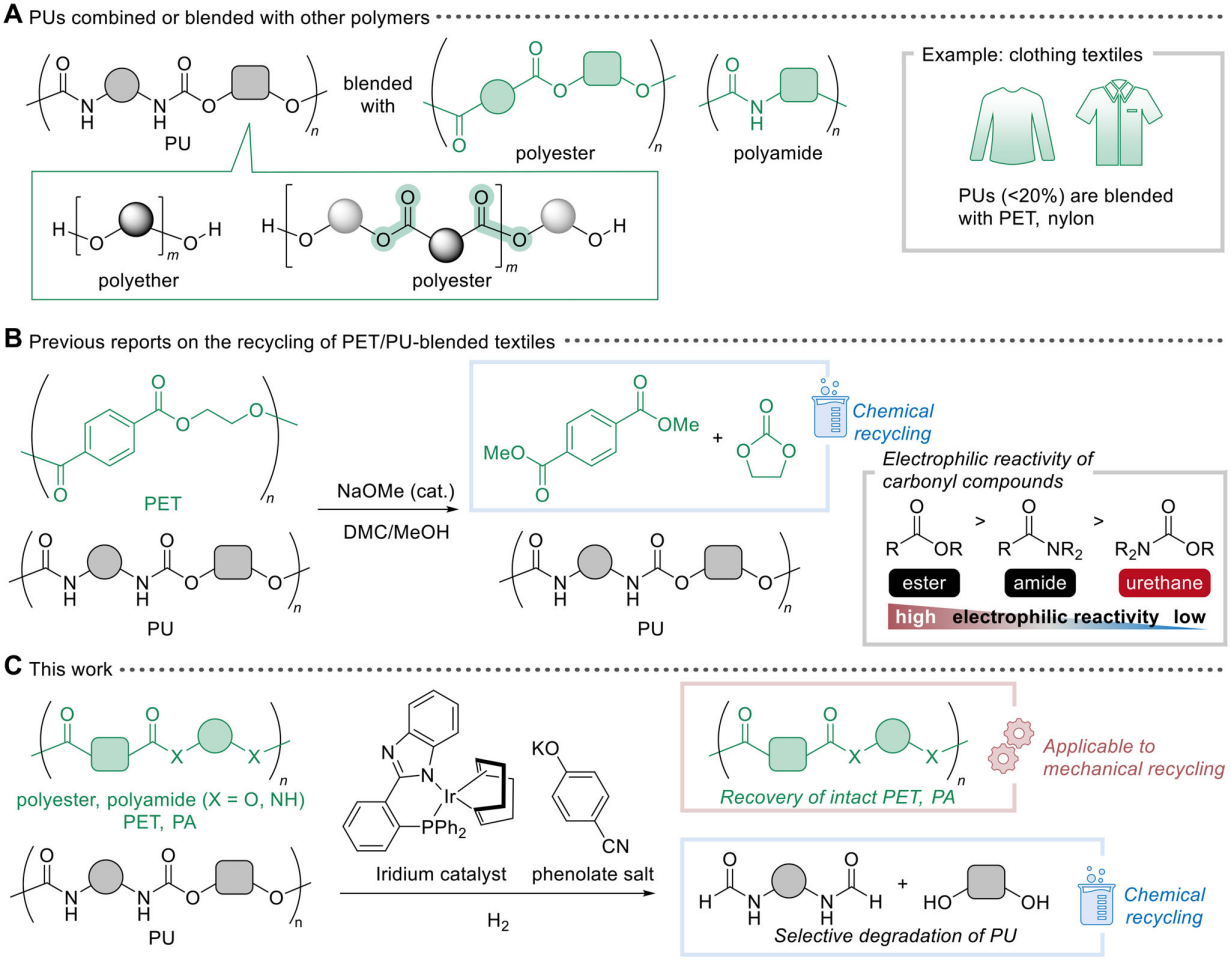
Recycling of polyurethanes in mixed plastic wastes. (A) PUs combined or blended with other polymers. (B) Previous reports on the recycling of PET/PU‐blended textiles [52]. (C) Selective hydrogenolysis of PUs to formamides and alcohols in mixed plastic waste using iridium catalyst and phenolate salt (This work).

Polyurethane (PU) is the sixth most widely used polymer and is renowned for its versatility, finding applications in various household items like mattresses, textiles, sponges, and mobile phone cases as well as car seats and building insulation [6, 7, 8]. Although some PUs are thermoplastics, most are thermosets, making mechanical recycling impractical and posing significant challenges for their recycling [9]. As a result, extensive research has focused on developing chemical degradation methods for urethane moieties [10], including glycolysis [11, 12, 13, 14], hydrolysis [15, 16, 17], acidolysis [18, 19, 20, 21, 22, 23, 24], aminolysis [25, 26], and hydrogenolysis [27, 28, 29, 30, 31, 32, 33, 34, 35, 36, 37, 38, 39, 40, 41]. These reactions, primarily involving nucleophilic substitution under harsh conditions due to the scarcity of electrophilicity of urethane bonds, may also react with other carbonyl‐containing compounds [42]. Notably, PUs are often combined or blended with other polymers, many of which contain carbonyl functionalities (Figure 1A). For instance, polyester‐based polyols, commonly derived from adipic acid and various diols, are frequently used in PU production to enhance mechanical strength [7]. In textiles, PUs are commonly blended as the flexible fiber Spandex (also known as elastane) with various fibers such as polyesters or nylons, typically in the range of 1%–20% [43, 44]. The demand for Spandex is projected to grow by 66% in the next decade, highlighting the urgent need for recycling technologies [45]. When applying the aforementioned degradation methods to these blended materials, both urethane bonds and ester and amide bonds can be cleaved, complicating the recovery of individual raw materials [44, 46]. To address these challenges, Li et al. [47, 48]. and we [49] have developed a catalytic hydrogenolysis of urethanes to formamides and alcohols, showing selective cleavage of urethane bonds while preserving more electrophilic other carbonyl functionalities with the counterintuitive chemoselectivity [50]. This transformation was also achieved using a heterogeneous Pd/Al_2_O_3_ catalyst [51]. To circumvent the difficulty of separating components in blended materials, selective degradation methods such as hydrolysis and methanolysis have been developed to depolymerize polyethylene terephthalate (PET) in PET/Spandex‐blended textiles by degrading only highly reactive ester bonds (Figure 1B) [52, 53]. While previous studies focused on the selective degradation of polyesters, PUs are often present in small proportions in such blends, as seen in PET/Spandex‐blended fibers [54]. Consequently, a common strategy for recycling Spandex‐blended textiles involves selectively dissolving Spandex using solvents for separate recovery [55]. However, this approach faces challenges due to limited research on repurposing dissolved Spandex, leading to its frequent disposal and focusing the recycling process on recovering less valuable PET and nylon while discarding the more valuable PU [56]. Recently, a new chemical recycling strategy has emerged to address this issue by directly depolymerizing PU components from blended textiles. Kristensen and Skrydstrup et al. developed a solvolysis approach using *tert*‐amyl alcohol and a catalytic amount of KOH to selectively disassemble Spandex fibers and PU coatings in textiles [57]. This method effectively depolymerizes PU from polyamide (nylon) blends while preserving the polyamide matrix. However, it lacks chemoselectivity when applied to polyester‐based fabrics, resulting in partial degradation of polyester fibers to recover fragmented polyesters. Similar changes in the molecular weight of polyesters due to transesterification have been observed in the selective hydrogenolysis of urethane, as reported by our group [49] and Kumar et al. [51]. This highlights a significant challenge: developing a chemical process that can robustly cleave stable urethane bonds without compromising the integrity of more reactive polymers like polyesters, particularly by suppressing side reactions such as reduction or transesterification. Therefore, the development of new chemical recycling methods capable of selectively degrading even minor components such as PUs while enabling material recovery of the remaining polymers presents a promising strategy for achieving energy‐efficient recycling of complex polymer blends.

In this study, we present a novel method for recycling PUs in conjunction with other carbonyl‐based polymers using iridium‐catalyzed selective hydrogenolysis. This process selectively degrades PUs while preserving other carbonyl‐containing polymers. An ongoing challenge in this area is the need to cleave the stable urethane bond, typically requiring harsh conditions such as the use of strong bases at temperatures exceeding 200°C in solvolysis [57]. These conditions often lead to the degradation of more delicate ester‐based polymers. Even at lower temperatures common in catalytic hydrogenolysis (120–150°C), the basic additives necessary for catalyst activation can promote undesirable transesterification [58], leading to the deterioration of polyester‐polyols and PET. To overcome these obstacles, we have devised a catalytic system that not only facilitates the selective hydrogenolysis of urethanes into formamides and alcohols but also effectively inhibits transesterification. This is achieved by combining the previously reported iridium catalyst [49, 59] with a phenolate salt additive (Figure 1C). This approach enables the recovery of intact carbonyl‐containing polymers, including polyesters, overcoming the primary limitations of current techniques. It also facilitates the energy‐efficient chemical recycling of complex polymer blends without the need for prior separation.

## 2 Results and Discussion

### 2.1 Condition Optimization

**FIGURE 2:**
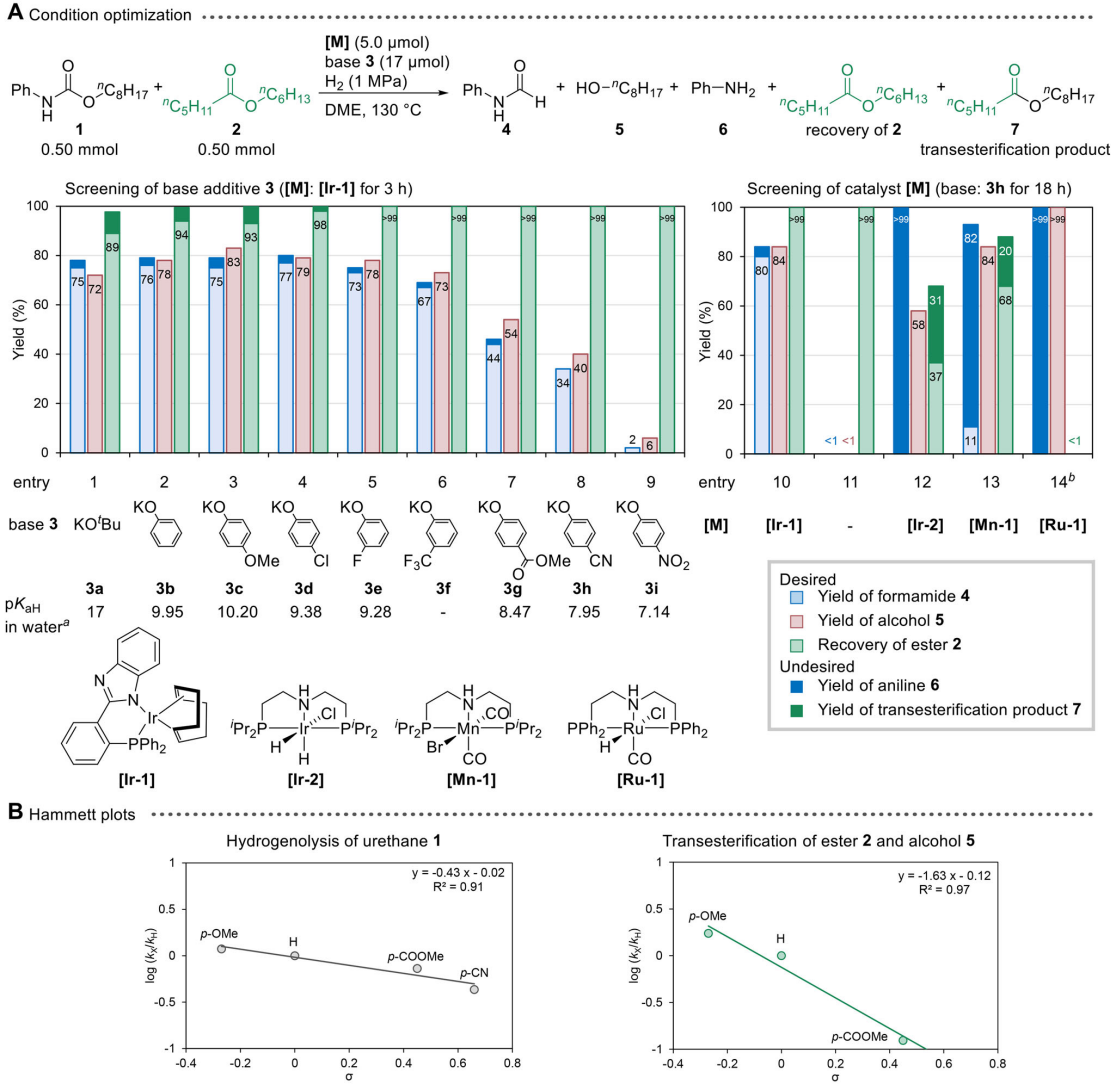
Condition optimization for selective hydrogenolysis of urethanes. (A) Condition optimization for selective hydrogenolysis of urethanes using various bases **3** and catalysts **[M]**. Reaction conditions: **1** (0.50 mmol), **2** (0.50 mmol), catalyst **[M]** (5.0 µmol) and base **3** (17 µmol) in 1,2‐dimethoxyethane (3 mL) under H_2_ (1 MPa) at 130°C. *a* p*K*_aH_ values for each base in water were taken from the literature [61]. *b* 4 MPa of H_2_. (B) Hammett plots for potassium phenolates bearing various substituents of hydrogenolysis of urethane **1** (left) and transesterification of ester **2** and alcohol **5** (right).

For the selective hydrogenative degradation of urethane in the presence of an ester, the combined use of an iridium hydrogenolysis catalyst and a base catalyst with appropriate basicity was found to be superior. To investigate the catalyst components and conditions, we first screened various base additives using a reaction mixture of octyl phenylcarbamate (**1**) and hexyl hexanoate (**2**) in the presence of an Ir complex **[Ir‐1]** and base **3** under hydrogen (1 MPa) in 1,2‐dimethoxyethane (DME) (for solvent screening, see Table S1) at 130°C for 3 h (Figure 2A). When KO*^t^* Bu (**3a**) was used, urethane **1** was rapidly hydrogenated to formamide **4** and alcohol **5**. However, subsequent transesterification between hexyl ester **2** and 1‐octanol (**5**) led to the formation of the unwanted octyl ester **7**, indicating that transesterification readily occurred under these conditions (Figure 2A, entry 1). Replacing **3a** with potassium phenolate (**3b**) promoted the hydrogenolysis of urethane **1** similarly; the yield of ester **7** was reduced, suggesting the partial suppression of transesterification (entry 2). Encouraged by these results, potassium phenolates bearing different substituents were explored. The potassium salt of the electron‐rich phenolate, potassium 4‐methoxyphenolate (**3c**), accelerated the formation of ester **7** (entry 3), whereas phenolates with electron‐withdrawing substituents (**3d–3h**) consistently suppressed the ester **7** formation (entries 4–8). Among them, potassium 4‐cyanophenolate (**3h**), bearing a strongly electron‐withdrawing group, completely suppressed transesterification while still enabling the hydrogenolysis of urethane **1** (entry 8). In contrast, potassium 4‐nitrophenolate (**3i**) with a stronger electron‐withdrawing group completely inhibited the hydrogenolysis of **1** (entry 9). Under the conditions in entry 8 (**3h** as the base), extending the reaction time to 18 h allowed sufficient conversion of **1** to **4** and **5**, along with minimal formation of ester **7**. This resulted in the selective hydrogenolysis of **1** and recovery of ester **2** (entry 10). In contrast, neither hydrogenolysis nor transesterification occurred without base additives (entry 11). Next, we examined other transition‐metal catalysts reported for the hydrogenolysis of urethanes [32] and esters [60].

The combination of Ir‐MACHO (**[Ir‐2]**) or Mn‐MACHO (**[Mn‐1]**) with potassium 4‐cyanophenolate (**3h**) led to the over‐reduction of formamide **4** to aniline **6** along with the hydrogenolysis of esters and significant transesterification (entries 12 and 13). Ru‐MACHO (**[Ru‐1]**), a well‐known catalyst for ester hydrogenolysis, was tested in the competitive reaction between urethane **1** and ester **2** at a high H_2_ pressure of 4 MPa, as reported previously [60]. The reaction resulted in the complete hydrogenolysis of both substrates, affording aniline **6** and 1‐octanol **5** via the cleavage of urethane **1** and two equivalents of 1‐hexanol from ester **2** (entry 14). These findings demonstrate that catalytic hydrogenolysis using **[Ir‐1]** and potassium 4‐cyanophenolate (**3h**) enable the chemoselective degradation of urethanes while effectively suppressing competing reactions such as ester reduction and transesterification.

The Hammett plots for both urethane hydrogenolysis and transesterification clearly illustrate the benefit of moderately electron‐withdrawing substituents on potassium phenolates for the selective degradation of urethanes over esters. Both reactions exhibited linear correlations between the log(*k*_X_/*k*_H_) and *σ* values [62] with negative *ρ* values. Importantly, the substantial difference in *ρ* values between transesterification (−1.63) and hydrogenolysis (−0.43) supports the conclusion that electron‐withdrawing substituents on phenolates preferentially inhibit transesterification without significantly hindering the hydrogenolysis of urethanes (Figure 2B).

### 2.2 Selective Degradation of Model Polyurethane

**FIGURE 3:**
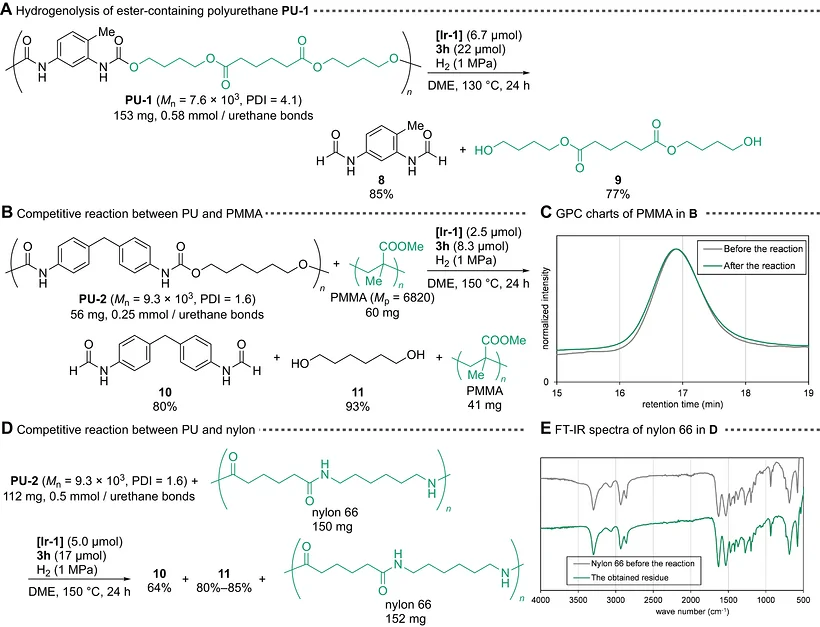
Selective degradation of model polyurethanes. (A) Hydrogenolysis of ester‐containing polyurethane **PU‐1**. (B) Competitive reaction between polyurethane **PU‐2** and PMMA. (C) GPC charts of PMMA before and after the reaction of **B** using RI detector. (D) Competitive reaction between polyurethane **PU‐2** and nylon 66. (E) FT‐IR spectra of nylon 66 before and after the reaction of **D**.

Under optimized conditions, we demonstrated the effectiveness of our catalytic system for selective hydrogenolysis of PU within a mixed polymer. First, we examined **PU‐1** (number average molecular weight (*M*_n_) = 7.6 × 10^3^, molecular‐weight dispersity (*M*_w_/*M*_n_) = 4.1), a PU derived from toluene‐2,4‐diisocyanate (2,4‐TDI) and an ester‐containing diol obtained from adipic acid and 1,4‐butanediol (Figure 3A). Consequently, the 2,4‐TDI‐based diformamide **8** (85%) and ester‐containing diol **9** (77%) were both obtained. This outcome highlights the potential of chemical recycling for PUs comprising polyester diols, allowing for the selective hydrogenolysis of urethane bonds while preserving the ester moieties (vide infra). Subsequently, we explored the competitive reaction involving polyurethane **PU‐2** (synthesized from methylenediphenyl 4,4′‐diisocyanate and 1,6‐hexanediol, *M*_n_ = 9.3 × 10^3^, *M*_w_/*M*_n_ = 1.6) and poly(methyl methacrylate) (PMMA) (peak top molecular weight (*M*_p_) = 6,820), another ester‐containing polymer. Blending PU with PMMA is a recognized approach to enhancing material mechanical properties [63, 64]. In line with these findings, the reaction selectively degraded the PU component, yielding diformamide **10** (80%) and diol **11** (93%) (Figure 3B). PMMA (41 mg) was separated via gel permeation chromatography (GPC) using tetrahydrofuran (THF) as the eluent. Comprehensive analyses, including GPC (Figure 3C), ^1^H nuclear magnetic resonance (NMR) spectroscopy, and Fourier transform infrared (FT‐IR) spectroscopy, confirmed that the recovered PMMA remained unchanged compared to the original sample (for additional analytical data, refer to Figure S4). A blend of **PU‐2** and nylon 66 was subjected to the optimized hydrogenolysis conditions (Figure 3D). This process effectively degraded **PU‐2**, yielding diformamide **10** and 1,6‐hexanediol (**11**) with NMR yields of 64% and 80%–85%, respectively (Figure S8), while a solid residue (152 mg) was retrieved. FT‐IR analysis of the residue indicated a spectrum identical to that of the original nylon 66, confirming the integrity of the polyamide (Figure 3E).

### 2.3 Application for Commercial Polyurethane Products

**FIGURE 4:**
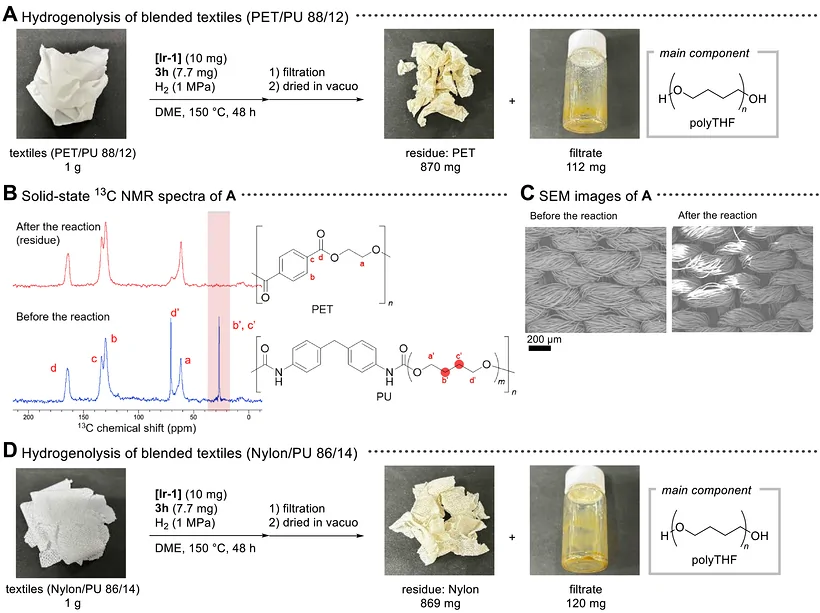
Selective degradation of polyurethanes in commercial clothing textiles. (A) Selective degradation of PUs in the commercial clothing textiles (PET/PU = 88/12). (B) Solid‐state ^13^C DDMAS NMR spectra of the textiles before and after the reaction **A**. (C) Scanning electron microscopy (SEM) observations of the textiles before and after the reaction **A**. (D) Selective degradation of PUs in the commercial clothing textiles (nylon/PU = 86/14).

The system was utilized for the selective degradation of PUs using technical‐grade materials. First, we targeted blended textiles comprising PET/PU and nylon/PU. While various methods for degrading blended textiles have been documented for polyester/PU [52, 53, 65], this study presents a new approach for selectively degrading low‐content PU components in both polyester/PU and nylon/PU textiles. A textile sample from a commercially available undershirt, consisting of polyester (PET) and PU in an 88/12 ratio, was tested (Figure 4A). Following treatment of the textile sample (1.0 g) with **[Ir‐1]**, **3h**, and H_2_ (1 MPa) at 150°C for 48 h, filtration of the resulting reaction mixture yielded a solid residue (870 mg) and filtrate (112 mg). Solid‐state ^13^C NMR analysis of the recovered residue confirmed the disappearance of PU, indicating the recovery of nearly pure PET (Figure 4B) [52]. Furthermore, scanning electron microscopy (SEM) analysis did not permit direct observation of the PU fibers even before treatment, possibly due to the low PU content in the original textile. However, the morphology of the recovered PET fibers remained unchanged post‐reaction (Figure 4C). Similarly, although the disappearance of PU‐derived signals could not be definitively confirmed by thermogravimetry‐differential thermal analysis (TG‐DTA) and FT‐IR due to the limited sensitivity of the minor component, the profiles obtained before and after treatment were almost identical, further suggesting the recovery of PET without structural degradation (Figures S6A,B). Moreover, ^1^H NMR analysis of the filtrate indicated the degradation of the PU component, yielding poly(tetrahydrofuran) (polyTHF) as the primary product (Figure S6C). GPC analysis of the filtrate indicated that polyTHF with an *M*_n_ = 1.5 × 10^4^ (polystyrene equivalent) was obtained (Figure S6D). Ethylene glycol, a primary monomer of PET, was not detected in the gas chromatography (GC) analysis of the filtrate (Figure S6E). These analyses demonstrated the successful degradation of PU into products primarily composed of polyTHF, facilitating the recovery of the PET component. Likewise, this approach was effectively applied to another mixed textile sourced from a commercially available undershirt: a blend of nylon and PU (86/14). The treatment led to the recovery of nylon fibers by selectively degrading the PU component (Figure 4D; for analytical data, refer to Figure S10).

**FIGURE 5:**
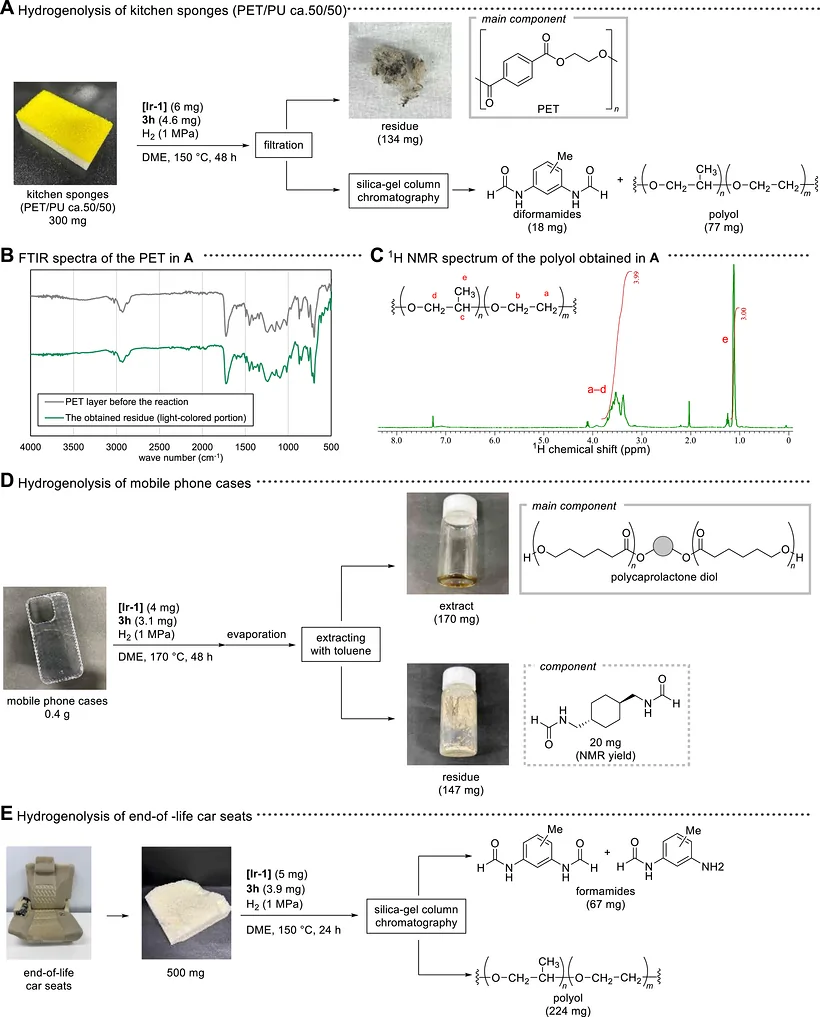
Selective hydrogenolysis of polyurethanes in commercial products. (A) Selective degradation of PUs in commercial kitchen sponges (PET/PU = ca. 50/50). (B) FT‐IR spectra of the PET before and after the reaction **A**. (C) ^1^H NMR spectrum of the polyol obtained in **A**. (D) Hydrogenolysis of commercial mobile phone cases. (E) Hydrogenolysis of end‐of‐life car seats.

Moreover, to demonstrate the applicability of the method beyond textiles, we utilized a commercially available kitchen sponge composed of PU foam and non‐woven PET fabric at a mass ratio of approximately 50:50 (Figure 5A). Following the reaction under the same conditions described above, the mixture underwent filtration to yield a solid residue (134 mg) and liquid filtrate (183 mg). The solid residue obtained contained a major light‐colored portion and a minor dark‐colored portion. FT‐IR analysis of the major light‐colored fraction exhibited a spectrum matching that of the PET layer before the reaction (Figure 5B). These results confirmed that the primary component of the recovered solid was PET, which remained intact without decomposition. Conversely, the filtrate underwent further purification via silica gel column chromatography to isolate the degradation products. This process resulted in a mixture of diformamides derived from 2,4‐ and 2,6‐toluenediamine (TDA) (18 mg) and the recovered polyol (77 mg). ^1^H NMR analysis of the polyol indicated a poly(propylene oxide) (PPO)/poly(ethylene oxide) (PEO) ratio of 80/20, consistent with the ratio of the commonly used PO‐EO copolyether polyol monomer (Figure 5C) [66]. GPC analysis revealed *M*_n_ = 4,000 and *M*_w_/*M*_n_ = 1.1 (Figure S10). Investigation was also conducted on commercial mobile phone cases composed of an ester‐containing polycaprolactone diol and aliphatic diisocyanate (1,4‐bis(isocyanatomethyl)cyclohexane) (Figure 5D). Following treatment using **[Ir‐1]**, **3h**, and H_2_ at 170°C for 48 h, and subsequent extraction using toluene, 170 mg of an extract primarily composed of polycaprolactone diol was obtained. Furthermore, ^1^H NMR and diffusion ordered spectroscopy (DOSY) analyses of the remaining solids suggested the presence of 20 mg of diformamide. (For the analytical data, refer to Figures S11–S14). This indicates that the catalytic reaction is also applicable to the degradation of PU derived from aliphatic diisocyanate monomers, and the ester groups in the polycaprolactone diol were not reduced. Finally, the focus shifted to the chemical recycling of end‐of‐life vehicles (ELVs).

The global generation of ELVs is estimated to be approximately 40 million units annually. Although legislation in many countries mandates recovery rates exceeding 95%, achieving this target necessitates advanced recycling technologies [67]. Plastics accounted for 15% of the total weight of ELVs, with PU being the second‐largest plastic component, constituting approximately 15% of this plastic fraction. Therefore, the development of effective PU recycling methods is of significant value [68]. To address this, the catalytic system was applied to ELV‐derived materials. Specifically, end‐of‐life car seats underwent the reaction, resulting in the successful isolation of a mixture of formamides (67 mg) and polyols (224 mg) using silica gel column chromatography. These results demonstrate that the proposed system effectively degrades PU in real‐world automotive waste, highlighting its potential for ELV recycling applications (Figure 5E; for analytical data, see Figures S16–S18).

## 3 Conclusion

We demonstrated the selective degradation of PUs in mixed plastic wastes, specifically those containing more reactive polyesters or polyamides, through hydrogenolysis catalyzed by an iridium complex and phenolate salts. Phenolate salt additives, particularly those with electron‐withdrawing groups, play a crucial role in promoting efficient hydrogenolysis of urethane bonds while effectively suppressing transesterification. This catalytic system enables the recovery of intact polyester and polyamide materials from polymer blends. The practical utility of this method is exemplified by its successful application in technical‐grade commercial products, including blended textiles for clothing, kitchen sponges, smartphone cases, and end‐of‐life car seats. In these applications, the PU component was selectively depolymerized, allowing for the isolation of polyester and nylon residues. This approach offers a distinct advantage over conventional methods by integrating material separation with specific depolymerization, thereby overcoming the challenges associated with recycling complex mixtures using the superior atomic and economic benefits of H_2_ as a reactant. Although addressing issues of cost‐effectiveness and scalability as well as isolation of degraded products will be a necessary future research, we anticipate that this system will serve as a novel chemical recycling strategy for mixed plastic waste, promoting composite separation and resource circulation in a circular economy.

## Author Contributions

**Yuto Yamada**: investigation, methodology, formal analysis, visualization, writing – original draft. **Takanori Iwasaki**: methodology, visualization, writing – original draft, conceptualization, supervision, funding acquisition, project administration. **Shinji Tanaka**: investigation, methodology, visualization, formal analysis, writing – review and editing. **Kyoko Nozaki**: supervision, resources, project administration, writing – review and editing, funding acquisition.

## Conflicts of Interest

The authors declare no conflicts of interest.

## Supporting information



The supporting information contains detailed condition screening, experimental procedures, and characterizations of degradation products. The authors have cited additional references within the Supporting Information [69–74].
**Supporting File**: anie73412‐sup‐0001‐SuppMat.pdf.

## Data Availability

The data that supports the findings of this study are available in the supplementary material of this article.
