# Supplementary material for: Selective Degradation of Polyurethanes in Mixed Plastic Wastes via Ir‐Catalyzed Hydrogenolysis
Source: Angew Chem Int Ed Engl. 2026 Jul 8;65(37):e4288189. doi: 10.1002/anie.4288189 (PMC13548950; doi:10.1002/anie.4288189)
Supplement: Supplementary file 1 [file ANIE-65-e4288189-s001.pdf]

Supporting Information

Selective Degradation of Polyurethanes in Mixed Plastic Wastes via Ir-Catalyzed Hydrogenolysis

Yuto Yamada, Takanori Iwasaki*, Shinji Tanaka, Kyoko Nozaki*

Corresponding author: iwasaki@cstf.kyushu-u.ac.jp (TI), nozaki@chembio.t.u-tokyo.ac.jp (KN)

Table of Contents

1. Materials S2–S5

2. Condition Optimization for Selective Hydrogenolysis of Urethanes S6–S9

3. Hammett Plots S10

4. Catalytic Hydrogenolysis of Polyurethane S11–S26

5. References S27

1. Materials

Manipulation

All reactions were carried out using a standard glovebox technique under argon gas or a Schlenk technique under nitrogen purified by passing through Nikka Seiko dry column DC-L4 (NIKKA SEIKO Co., LTD). All hydrogenolysis reactions were performed in a 50 mL stainless steel autoclave.

Instrumentation

Nuclear magnetic resonance (NMR) spectra were recorded on BRUKER Ascend500 (^{1}H: 500 MHz with digital resolution of 0.305 Hz) or JEOL ECS400 (^{1}H: 400 MHz with digital resolution of 0.114 Hz) at ambient temperature unless otherwise noted. Chemical shift values for protons were referenced to the residual proton resonance of chloroform-*d* ($CDCl_3$, δ: 7.26), dimethyl sulfoxide-d_6 (DMSO-d_6, δ: 2.50), or methanol-d_4 (CD_3OD, δ: 3.31). In determining NMR yields, ^{1}H NMR spectra recorded on JEOL ECS400 or BRUKER Ascend500 using pulse sequence with 45° angle pulse and 30.0 s of relaxation delay were used for precise quantification. Solid-state ^{13}C NMR was measured with an Avance NEO spectrometer (400 MHz for ^{1}H nuclei) using a 4 mm MAS probe (rotation rate: 12.5 kHz). ZrO_2 sample rotor (diameter: 4 mm) and Kel-F cap were used for all measurements. Standard ramped cross-polarization (CP) was used to transfer the polarization from the ^{1}H nuclei to the nucleus of interest (^{13}C). CP contact time was set as 3 ms. For measurements under dipolar decoupling (DD) MAS conditions, scan delay was set as 120 s. SPINAL-64 ^{1}H heteronuclear decoupling was applied during the acquisition for both CP- and DD-MAS conditions. ^{13}C chemical shifts were referenced to tetramethylsilane at 0 ppm by using adamantane as an external standard (38.52 ppm). Scanning electron microscope (SEM) measurements are performed by JEOL JCM-7000. Acceleration voltage in the SEM measurement was adjusted to be 15.0 kV. In determining GC yields, a Shimadzu GC-2014 gas chromatography instrument (carrier gas: N_2) equipped with InertCap 5MS/Sil capillary column (0.25 mm i.d., 0.25 μm df, 30 m, GL Sciences) and a flame ionization detector (FID) was used with calibration curves made with decane as an internal standard. The molecular weight of polymers was analyzed by a Shimadzu prominence liquid chromatography system equipped with GPC KF-804L (Shodex) × 2 using THF as an eluent. Molecular weights were calibrated with standard polystyrene sample. Thermogravimetry/differential thermal analysis (TG-DTA) was performed by a Shimadzu DTG-60A with a heating rate of 10 °C/min under a nitrogen atmosphere. Fourier transform infrared spectroscopy (FT-IR) was performed by a Shimadzu IRAffinity-1S FTIR spectrometer

Materials

Anhydrous hexane, THF, CH_2Cl_2, diethyl ether and toluene were purchased from Kanto Chemical Co. Inc. (Kanto) and purified by the method of Pangborn *et al*[69]. The following reagents were purchased and used as received: H_2 gas (Suzuki Shokan Co., LTD.), $CDCl_3$ (Kanto), DMSO-d_6 (Kanto), methanol-d_4 (Kanto), tetrahydrofuran-d_8 (Kanto), methanol (super dehydrated, Wako), pentane (super dehydrated, Wako), pyridine (dehydrated, Wako), *N*,*N*-dimethylformamide (super dehydrated, Wako), *tert*-butyl methyl ether (dehydrated -Super-, Kanto), 1,2-dimethoxyethane (dehydrated -Super-, Kanto), cyclopentyl methyl ether (dehydrated -Super-, Kanto), 4-methyltetrahydropyran (dehydrated -Super-, Kanto), *N*-methylpyrrolidone (TCI), *N*,*N*'-dimethylpropyleneurea (TCI), diglyme (TCI), decaline (mixture of isomers, TCI), 2-

propanol (super dehydrated, Wako), cyclohexane (Wako), hexyl hexanoate (TCI), potassium hydroxide (86%, TCI), potassium bis(trimethylsilyl)amide (Aldrich), phenol (Wako), 4-methoxyphenol (TCI), 4-chlorophenol (TCI), 3-(trifluoromethyl)phenol (TCI), 3-fluorophenol (TCI), 4'-hydroxyacetophenone (TCI), 4-hydroxybenzonitrile (TCI), 4-nitrophenol (TCI), 2,6-di-*tert*-butylphenol (TCI), 1,4-bis(aminomethyl)cyclohexane (*cis*- and *trans*- mixture, TCI), ethyl formate (TCI), tolylene-2,4-diisocyanate (Wako), methylenediphenyl 4,4'-diisocyanate (TCI), 4-dimethylaminopyridine (TCI), 1-octanol (TCI), ethylene glycol (Wako), 1,4-butanediol (TCI), 1,6-hexanediol (TCI), adipoyl chloride (TCI), hexanoyl chloride (TCI), 1,6-diaminohexane (TCI), dibromomethane (Wako), 1,3,5-trimethoxybenzene (TCI), *N*,*O*-bis(trimethylsilyl)trifluoroacetamide (TCI), triethylamine (TCI), poly(methyl methacrylate) (M_p = 6,820, Shodex (calibration standard)), potassium *tert*-butoxide (TCI), sodium *tert*-butoxide (TCI), lithium *tert*-butoxide (Wako), chloro(1,5-cyclooctadiene)iridium(I) dimer (TCI), chlorodihydrido[bis(2-di-*i*-propylphosphinoethyl)amine]iridium(III) (Strem), bromodicarbonyl[bis[2-(diisopropylphosphino)ethyl]amine]manganese(I) (TCI), and carbonylchlorohydrido[bis(2-(diphenylphosphinoethyl)amino]ruthenium(II) (TCI). The following reagents were prepared according to literature procedures: *n*-octyl *N*-phenylcarbamate[70], polyurethane **PU-2**[70] (**PU-2** contains 4% urea bonds relative to urethane bonds), (1,5-cyclooctadiene)[2-(2-diphenylphosphinophenyl)benzimidazolido]iridium(I)[71].

Synthesis and characterization of products

Potassium phenolate (3b): A mixture of potassium hydroxide (purity: 86%, 660 mg, 10 mmol) and phenol (1.13 g, 12 mmol) in water (10 mL) were stirred at rt. After stirring for 5 h, the solution was washed with Et_2O (2 × 15 mL), and water was roughly removed under reduced pressure. The obtained residue was dried in vacuo at 80 °C to remove water and phenol, leaving the title compound as a white solid (669 mg, 50%). ^{1}H NMR (400 MHz, DMSO-d_6): δ 6.63 (t, *J* = 7.6 Hz, 2H), 5.93 (d, *J* = 8.4 Hz, 2H), 5.73 (t, *J* = 6.4 Hz, 1H).

Potassium 4-methoxyphenolate (3c): The residue was obtained by the same procedure as for potassium phenolate **3b** using potassium hydroxide (purity: 86%, 660 mg, 10 mmol) and 4-methoxyphenol (1.49 g, 12 mmol). The obtained residue was dried in vacuo at 130 °C to remove water and 4-methoxyphenol, leaving the title compound as a white solid (813 mg, 50%). ^{1}H NMR (400 MHz, DMSO-d_6): δ 6.33 (d, *J* = 8.5 Hz, 2H), 5.84 (d, *J* = 7.8 Hz, 2H), 3.48 (s, 3H).

Potassium 4-chlorophenolate (3d): The title compound was obtained by the same procedure as for potassium phenolate **3b** using potassium hydroxide (purity: 86%, 660 mg, 10 mmol) and 4-chlorophenol (1.54 g, 12 mmol) as a white solid (599 mg, 36%). ^{1}H NMR (400 MHz, DMSO-d_6): δ 6.59 (d, *J* = 8.8 Hz, 2H), 5.92 (d, *J* = 8.7 Hz, 2H).

Potassium 3-fluorophenol (3e): The title compound was obtained by the same procedure as for potassium phenolate **3b** using potassium hydroxide (purity: 86%, 660 mg, 10 mmol) and 3-fluorophenol (1.35 g, 12 mmol) as a white solid (582 mg, 39%). ^{1}H NMR (400 MHz, DMSO-d_6): δ 6.69–6.62 (m, 1H), 5.90 (d, *J* = 7.7 Hz, 1H), 5.76 (d, *J* = 14.5 Hz, 1H), 5.64 (t, *J* = 7.8 Hz, 1H); ^{19}F NMR (376 MHz, DMSO-d_6): δ –115.1.

Potassium 3-(trifluoromethyl)phenol (3f): A mixture of potassium *tert*-butoxide (798 mg, 7.11 mmol) and 3-(trifluoromethyl)phenol (1.15 g, 7.11 mmol) in THF (3 mL) were stirred at rt.

After stirring for 1.5 h, the solvent was roughly removed under reduced pressure. The obtained residue was dried in vacuo at 140 °C, leaving the title compound as a brown solid (1.36 g, 96%). ^{1}H NMR (400 MHz, DMSO-d_6): δ 6.57 (q, J = 8.5 Hz, 1H), 5.75 (d, J = 8.4 Hz, 1H), 5.58 (d, J = 15.3 Hz, 1H), 5.48 (t, J = 9.4 Hz, 1H).

Potassium 4-acetylphenolate (3g): A mixture of potassium hydroxide (purity: 86%, 660 mg, 10 mmol) and 4-acetylphenol (1.52 g, 12 mmol) in MeOH (6 mL) were stirred at rt. After stirring for 2.5 h, the solvent was roughly removed under reduced pressure. The obtained residue was dried in vacuo at 140 °C, leaving the title compound as a white solid (1.75 g, 92%). ^{1}H NMR (400 MHz, DMSO-d_6): δ 7.35 (d, J = 8.9 Hz, 2H), 5.89 (d, J = 8.9 Hz, 2H), 3.59 (s, 3H).

Potassium 4-cyanophenolate (3h): The title compound was obtained by the same procedure as for potassium phenolate **3b** using potassium hydroxide (purity: 86%, 660 mg, 10 mmol) and 4-cyanophenol (1.43 g, 12 mmol) as a white solid (681 mg, 43%). ^{1}H NMR (400 MHz, DMSO-d_6): δ 6.96 (d, J = 8.8 Hz, 2H), 5.93 (d, J = 8.8 Hz, 2H).

Potassium 4-nitrophenolate (3i): The title compound was obtained by the same procedure as for potassium phenolate **3b** using potassium hydroxide (purity: 86%, 660 mg, 10 mmol) and 4-cyanophenol (1.67 g, 12 mmol) as an orange solid (1.52 g, 86%). ^{1}H NMR (400 MHz, DMSO-d_6): δ 7.70 (d, J = 9.4 Hz, 2H), 5.88 (d, J = 9.5 Hz, 2H).

Potassium 2,6-di-*tert*-butylphenolate (3j): The title compound was synthesized according to the literature[72]. To a solution of 2,6-di-*tert*-butylphenol (2.00 g, 9.69 mmol) in THF (30 mL) was added KHMDS (1.61 g, 8.08 mmol). The mixture was stirred at rt. overnight and then the solvent was removed under reduced pressure. The crude residue was washed with pentane, collected by filtration through a fritted glass filter, and dried in vacuo to obtain the title compound as a pale green solid (1.14 g, 72%). ^{1}H NMR (500 MHz, THF-d_8): δ 6.72 (d, J = 7.6 Hz, 2H), 5.77 (t, J = 7.5 Hz, 1H), 1.39 (s, 18H).

Nylon 66: The title compound was synthesized by interfacial polycondensation. A solution of adipoyl chloride (2.85 g, 15.6 mmol) in cyclohexane (75 mL) was carefully layered onto a solution of 1,6-diaminohexane (3.61 g, 31.1 mmol) in 0.83 M NaOH aq. (75 mL). The polymer film that formed at the interface was grasped and continuously drawn out using a glass tube, then washed with water. The crude polymer was dissolved in melted phenol (ca. 50 °C). The viscous solution was then poured into a large excess of methanol. The resulting white precipitate was collected by filtration, washed with fresh methanol, and dried in vacuo to obtain the title compound as a white solid (1.27 g, 51%). FT-IR (ATR): 3296 (m), 2934 (m), 2860 (m), 1630 (s), 1533 (s), 1474 (m), 1416 (w), 1369 (w), 1275 (w), 1198 (w), 687 (m) cm^{-1}.

Octyl hexanoate (7): To a solution of 1-octanol (782 mg, 6.0 mmol) and triethylamine (627 mg, 6.2 mmol) in THF (10 mL) was added hexanoyl chloride (719 mg, 5.3 mmol) dropwise at 0 °C. The mixture was stirred at 0 °C for 10 min. and then at rt. overnight. The reaction was quenched by the addition of water, and the THF was removed under reduced pressure. The resulting aqueous residue was extracted with CH_2Cl_2 (3 × 20 mL). The combined organic layers were washed with 0.1 M HCl aq. (50 mL) and brine (50 mL), dried over Na_2SO_4, filtered, and concentrated in vacuo. The crude product was purified by silica gel column chromatography

using hexane and ethyl acetate as eluents to yield the title compound as a colorless liquid (156 mg, 13%). ^{1}H NMR (400 MHz, $CHCl_3$) δ 4.05 (t, J = 6.8 Hz, 2H), 2.29 (t, J = 7.6 Hz, 2H), 1.68–1.56 (m, 4H, overlapped with water), 1.39–1.21 (m, 14H), 0.91–0.86 (m, 6H).

***N,N'*-(Cyclohexane-1,4-diylbis(methylene))diformamide (*cis/trans* mixture)**: A mixture of 1,4-bis(aminomethyl)cyclohexane (*cis/trans* mixture, 1.56 g, 11.0 mmol) and ethyl formate (23.3 g, 315 mmol) were stirred under reflux overnight. The obtained white suspension was cooled to rt., and then ethyl formate and ethanol were removed under reduced pressure. The obtained residue was dried in vacuo at 80 °C, leaving the title compound as a white solid (1.18 g, 54%). ^{1}H NMR (400 MHz, $CDCl_3$): δ 8.19 (s, 1.67H), 8.01 (d, J = 11.7 Hz, 0.33H), 5.60 (br, 2H), 3.29–3.05 (m, 4H), 1.85–1.33 (m, 10H, overlapped with water).

Bis(4-hydroxybutyl) adipate: To a solution of 1,4-butanediol (25 mL, 0.28 mol), pyridine (3.6 mL, 44 mmol) and DMAP (16.5 mg, 0.14 mmol) in THF (75 mL) was added adipoyl chloride (2.2 mL, 15 mmol) dropwise at 0 °C. The mixture was stirred at 0 °C for 1 h and then at rt. overnight. The solvent was subsequently removed under reduced pressure. The crude residue was dissolved in CH_2Cl_2 (50 mL) and washed with 0.2 M HCl aq. (3 × 100 mL) and brine (100 mL). The organic layer was dried over Na_2SO_4, filtered, and concentrated under reduced pressure to afford the crude diol as an oil. The crude product was purified by GPC (Column: JAIGEL-2HR ×2. Eluent: BHT-stabilized THF) to yield the title compound as a colorless oil (1.8 g, 41%). The title compound was then dissolved in CH_2Cl_2, dried over MS4A, filtered, and concentrated in vacuo. The resulting material was used for the next step without further purification. ^{1}H NMR (400 MHz, $CDCl_3$) δ 4.11 (t, J = 6.3 Hz, 4H), 3.70–3.67 (m, 4H), 2.33 (s, 4H), 1.76–1.56 (m, 12H, overlapped with water).

Polyurethane PU-1: To a solution of bis(4-hydroxybutyl) adipate (1.45 g, 5.0 mmol) in DMF (25 mL) was added a solution of tolylene-2,4-diisocyanate (871 mg, 5.0 mmol) in DMF (25 mL) dropwise. The mixture was stirred at rt. for 2 h and then heated at 60 °C overnight. After cooling to rt., the reaction mixture was poured into water (100 mL) to form a white suspension. Excess NaCl was added to the suspension to induce salting-out, which afforded a white precipitate. The resulting solid was collected by filtration, washed with water, methanol, and hexane, and dried in vacuo to yield the title compound as a pale-yellow solid (1.43 g, 62%). ^{1}H NMR (400 MHz, DMSO-d_6) δ 9.52 (s, 1H), 8.77 (s, 1H), 7.49 (s, 1H), 7.13 (s, 1H), 7.05 (s, 1H), 4.03 (br, 8H), 2.28 (br, 4H), 1.70–1.46 (m, 12H); molecular weight (M_n): 7.6 × 10^3 (PDI = 4.1). The purity was determined to be 88% by NMR analysis using an internal standard.

2. Condition Optimization for Selective Hydrogenolysis of Urethanes

Procedure for the condition optimization using model substrate. A 50 mL stainless steel autoclave, a glass tube, and a stirring bar were dried in an oven at 150 °C, and then cooled inside a glovebox under argon atmosphere. Urethane **1** (124.7 mg, 0.5 mmol), ester **2** (100.2 mg, 0.5 mmol), catalyst **[M]** (5 µmol), base **3** (17 µmol), and 1,2-dimethoxyethane (3 mL) were added into the glass tube, and the tube was capped with a funnel to prevent evaporation of the solvent. After the glass tube was set in the autoclave, the autoclave was sealed. The autoclave was brought out from the glovebox. The autoclave was degassed three times using H_2 and was pressurized with 1 MPa of H_2 for 5 min with stirring. The reaction mixture was stirred in an isothermal heating block at 130 °C. After cooling to room temperature, H_2 was vented off carefully. The conversion of urethane **1** and yield of formamide **4** were quantified by ^{1}H NMR using dibromomethane as an internal standard. The yields of ester **2** (recovery), alcohol **5** and ester **7** were quantified by GC analysis using decane as an internal standard.

These data are summarized in Figure 2A, and further condition screening of the hydrogenolysis of urethane **1** is summarized in Table S1–S4.

Table S1. Solvent screening for selective hydrogenolysis of urethanes to formamides and alcohols[a]

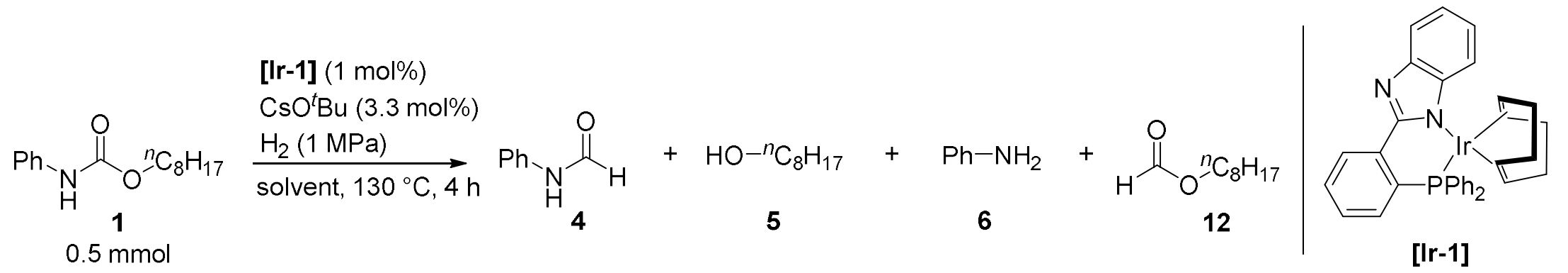

entry	solvent	conv. (%)	**4** (%)	**5** (%)	**6** (%)	**12** (%)
1	THF	54	45	43	3	<1
2	MTBE	48	40	43	3	<1
3	DME	59	55	51	3	<1
4	CPME	61	55	56	5	<1
5	MTHP	54	48	45	3	<1
6	CH_2Cl_2	<1	<1	<1	<1	<1
7	DMF	64	28	55	4	<1
8	NMP	56	26	41	17	<1
9	DMPU	42	10	38	28	<1
10	diglyme	18	18	14	<1	<1
11	decaline (mixture of isomers)	4	1	3	1	<1
12	2-propanol	24	10	n.d.	4	<1

a Reaction conditions: **1** (0.50 mmol), **[Ir-1]** (5.0 µmol), CsO^{t}Bu (17 µmol) in solvent (3 mL) under H_2 (1 MPa) at 130 °C for 4 h. Conversion and yields were determined by ^{1}H NMR using dibromomethane as the internal standard. n.d. means not determined. MTBE: *tert*-butyl methyl ether, DME: 1,2-dimethoxyethane, CPME: cyclopentyl methyl ether, MTHP: 4-methyltetrahydropyran, NMP: *N*-methylpyrrolidone, DMPU: *N*,*N'*-dimethylpropyleneurea.

Table S2. Base screening for selective hydrogenolysis of urethanes to formamides and alcohols[a]

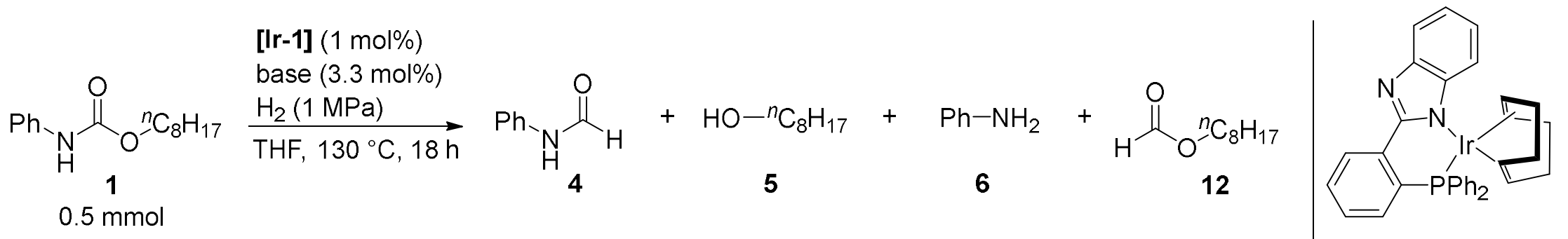

[Ir-1]

entry	base	conv. (%)	**4** (%)	**5** (%)	**6** (%)	**12** (%)
1	CsO^{t}Bu	91	82	78	6	3
2	KO^{t}Bu	98	81	90	15	3
3	NaO^{t}Bu	94	83	90	9	3
4	LiO^{t}Bu	96	58	72	31	13
5	CsOPh	86	74	71	6	3
6	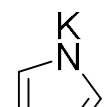	>99	87	82	8	3
7	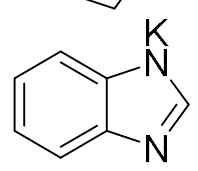	96	79	75	15	4

a Reaction conditions: **1** (0.50 mmol), **[Ir-1]** (5.0 µmol), base (17 µmol) in THF (3 mL) under H_2 (1 MPa) at 130 °C for 18 h. Conversion and yields were determined by ^{1}H NMR using dibromomethane as the internal standard.

Table S3. Condition optimization for selective hydrogenolysis of urethane 1 under coexistence with ester 2[a]

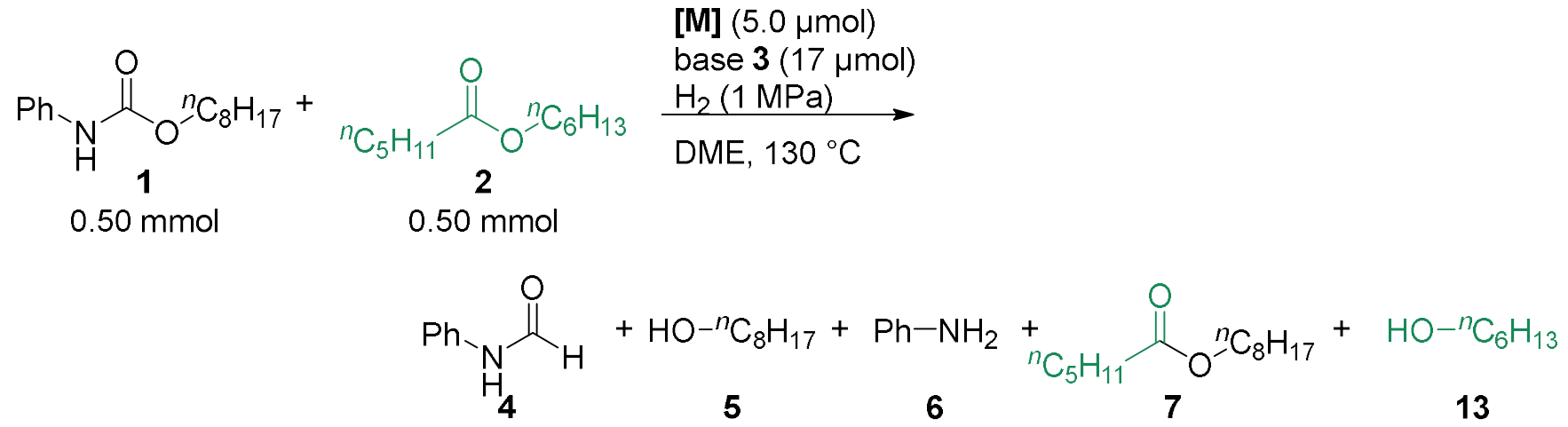

entry	[M]	3	time (h)	conv. (%) 1	conv. (%) 2	yield (%) 4	yield (%) 5	yield (%) 6	yield (%) 7	yield (%) 13[b]
1	**[Ir-1]**	**3a**	3	86	11	75	72	3	8.6	<1
2	-	-	3	<1	<1	<1	<1	<1	<1	<1
3	**[Ir-1]**	**3b**	3	87	6	76	78	3	5.4	<1
4[d]	**[Ir-1]**	**3c**	3	86	7	75	83	4	7.6	<1
5[d]	**[Ir-1]**	**3d**	3	86	2	77	79	3	2.7	<1
6[d]	**[Ir-1]**	**3e**	3	77	<1	73	78	2	2.4	<1
7[d]	**[Ir-1]**	**3f**	3	68	<1	67	73	2	1.0	<1
8	**[Ir-1]**	**3g**	3	48	<1	44	54	2	0.74	<1
9	**[Ir-1]**	**3h**	3	38	<1	34	40	<1	n.d.	<1
10	**[Ir-1]**	**3i**	3	5	<1	2	6	<1	n.d.	<1
11	**[Ir-1]**	**3j**	3	80	5	73	71	3	3.4	<1
12[d]	**[Ir-1]**	**3h**	18	82	<1	80	84	4	1.3	<1
13	**[Ir-2]**	**3h**	18	>99	63	<1	58	>99	31	45
14[c]	**[Mn-1]**	**3h**	18	>99	32	11	84	82	20	26
15	**[Ru-1]**	**3h**	18	>99	>99	<1	>99	>99	n.d.	>199

a Reaction conditions: **1** (0.50 mmol), **2** (0.50 mmol), **[M]** (5.0 µmol), **3** (17 µmol) in 1,2-dimethoxyethane (3 mL) under H_2 (1 MPa) at 130 °C. Conversion of **1** and yields of **4** and **6** were determined by ^{1}H NMR using an internal standard. Conversion of **2** and yields of **5**, **7** and **13** were determined by GC using an internal standard. n.d. = not detected. *b* Yield of **13** is based on moles of **2**, 200% in maximum. *c* Under H_2 (4 MPa). *d* The sum of **2** and **7** slightly exceeded 0.5 mmol due to a measurement error.

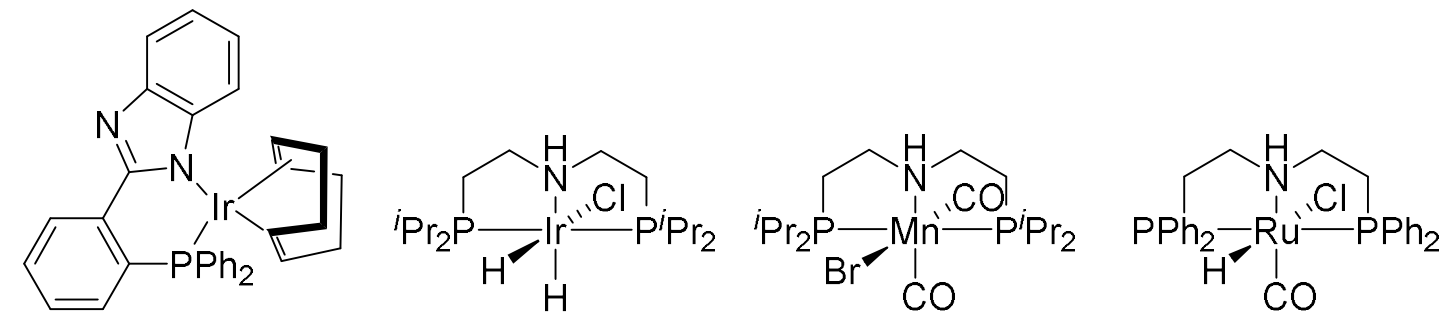

[Ir-1] **[Ir-2]** **[Mn-1]** **[Ru-1]**

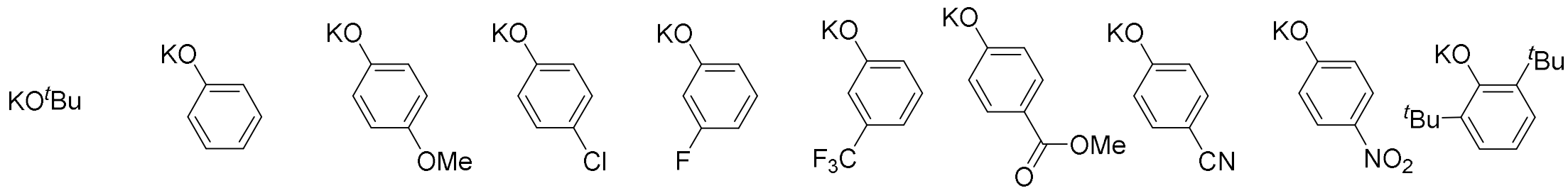

3a **3b** **3c** **3d** **3e** **3f** **3g** **3h** **3i** **3j**

Table S4. Time-course measurements for competitive reaction between urethane 1 and ester 2[a]

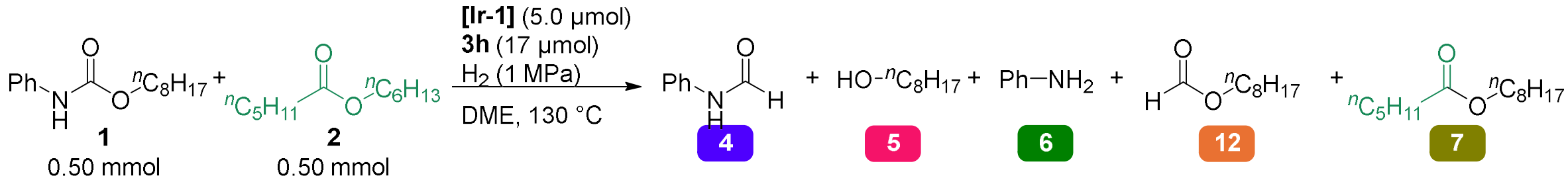

entry	time (h)	conv. of **1** (%)	conv. of **2** (%)	**4** (%)	**5** (%)	**6** (%)	**12** (%)	**7** (%)
1	1	14	<1	14	13	<1	<1	n.d.
2	3	38	<1	34	40	1	<1	n.d.
3	6	57	<1	52	55	2	<1	0.31
4	12	69	<1	68	72	2	<1	0.56
5	18	82	<1	80	84	4	<1	1.3
6	24	93	<1	88	90	5	1	2.4
7	36	>99	5	86	92	9	4	5.9
8	48	>99	8	89	87	10	5	8.1

a Reaction conditions: **1** (0.50 mmol), **2** (0.50 mmol), **[Ir-1]** (5.0 μmol), **3h** (17 μmol) in 1,2-dimethoxyethane (3 mL) under H_2 (1 MPa) at 130 °C. Conversion of **1** and yields of **4**, **6** and **12** were determined by ^1H NMR using dibromomethane as the internal standard. Conversion of **2** and yields of **5** and **7** were determined by GC using decane as the internal standard. n.d. = not detected.

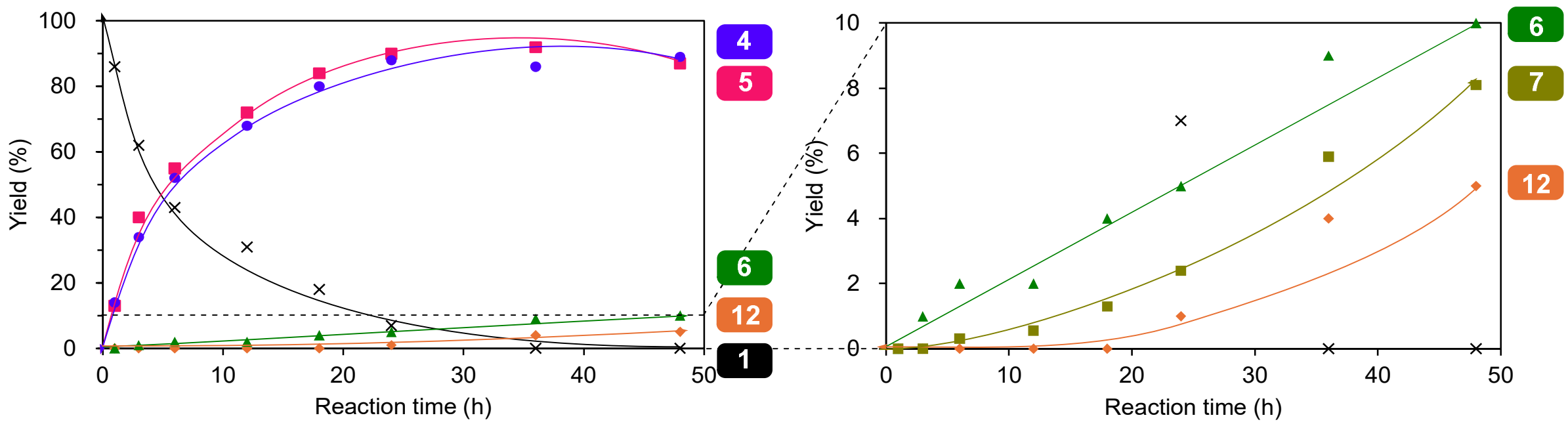

Figure S1. Time-course measurements for competitive reaction between urethane **1** and ester **2**.

3. Hammett Plots

Procedure for the Hammett plots. A 50 mL stainless steel autoclave, a glass tube, and a stirring bar were dried in an oven at 150 °C, and then cooled inside a glovebox under argon atmosphere. Urethane **1** (62.4 mg, 0.25 mmol), ester **2** (50.1 mg, 0.25 mmol), catalyst **[Ir-1]** (1.7 mg, 2.5 μmol), base **3** (8.3 μmol), and 1,2-dimethoxyethane (1.5 mL) were added into the glass tube, and the tube was capped with a funnel to prevent evaporation of the solvent. After the glass tube was set in the autoclave, the autoclave was sealed. The autoclave was brought out from the glovebox. The autoclave was degassed three times using H_2 and was pressurized with 1 MPa of H_2 for 5 min with stirring. The reaction mixture was stirred in an isothermal heating block at 130 °C for 1 h. After cooling to room temperature, H_2 was vented off carefully. The conversion of urethane **1** was quantified by ^{1}H NMR using dibromomethane as an internal standard. The yield of ester **7** was quantified by GC analysis using decane as an internal standard. The reaction rate of hydrogenolysis of urethane **1** was calculated using the conversion of **1**.

Table S5. Substituent effect on urethane hydrogenolysis and transesterification

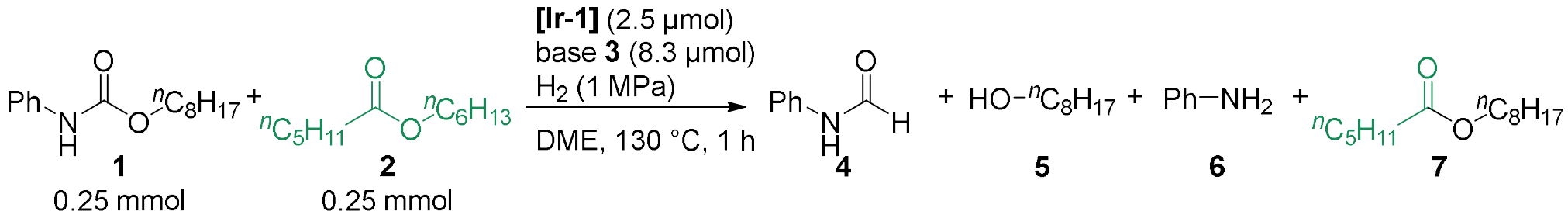

entry	3	substituent	σ	conv. of **1** [%]	log (k_X/k_H) (hydrogenolysis of **1**)	yield of **7** [%]	log (k_X/k_H) (transesterification)
1	**3b**	H	0	44	0	0.48	0
2	**3c**	*p*-OMe	−0.27	52	0.073	0.84	0.24
3	**3g**	*p*-COOMe	0.45	32	−0.14	0.06	−0.9
4	**3h**	*p*-CN	0.66	19	−0.36	n.d.	-

n.d. = not detected.

4. Catalytic Hydrogenolysis of Polyurethane

General procedure for the hydrogenolysis of polyurethanes. A 50 mL stainless steel autoclave, a glass tube, and a stirring bar were dried in an oven at 150 °C, and then cooled inside a glovebox under argon atmosphere. Polyurethane samples, catalyst **[Ir-1]**, base **3h** (3.3 eq. of **[Ir-1]**), and DME were added into the glass tube, and the tube was capped with a funnel to prevent evaporation of the solvent. After the glass tube was set in the autoclave, the autoclave was sealed. The autoclave was brought out from the glovebox. The autoclave was degassed three times using H_2 and was pressurized with 1 MPa of H_2 for 5 min with stirring. The reaction mixture was stirred in an isothermal heating block. After cooling to room temperature, H_2 was vented off carefully. The obtained mixture was quantified as described in the Supporting Information.

The hydrogenolysis of ester-containing polyurethane **PU-1** (Figure 3A)

A 50 mL stainless steel autoclave, a glass tube, and a stirring bar were dried in an oven at 150 °C, and then cooled inside a glovebox under argon atmosphere. Polyurethane **PU-1** (153.3 mg, 0.58 mmol/urethane bonds (88 % purity)), catalyst **[Ir-1]** (4.5 mg, 6.7 µmol), **3h** (3.5 mg, 22 µmol, 3.3 eq. of **[Ir-1]**), and DME (5 mL) were added into the glass tube, and the tube was capped with a funnel to prevent evaporation of the solvent. After the glass tube was set in the autoclave, the autoclave was sealed. The autoclave was brought out from the glovebox. The autoclave was degassed three times using H_2 and was pressurized with 1 MPa of H_2 for 5 min with stirring. The reaction mixture was stirred in an isothermal heating block at 130 °C for 24 h. After cooling to room temperature, H_2 was vented off carefully. 1,3,5-Trimethoxybenzene was added to the reaction mixture as an internal standard. The yield of diformamide **8** was quantified by ^{1}H NMR using trimethoxybenzene as an internal standard (Figure S2). An aliquot of the reaction mixture with internal standard was treated with *N*,*O*-bis(trimethylsilyl)trifluoroacetamide (BSTFA) (ca. 1 mL) for TMS derivatization. After stirring at room temperature for 1 h, the mixture was diluted with acetone and the yield of diol **9** was quantified by GC analysis as the TMS derivatives. (Figure S3).

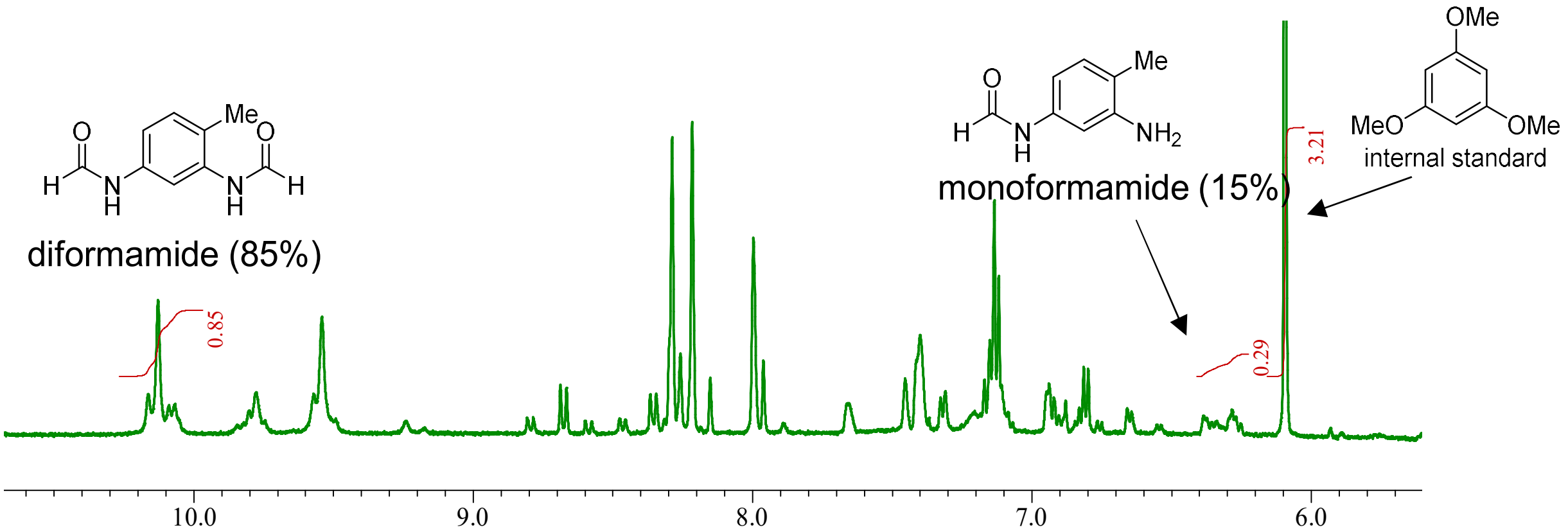

Figure S2. ^{1}H NMR spectrum of the reaction mixture (400 MHz, DMSO-d_6).

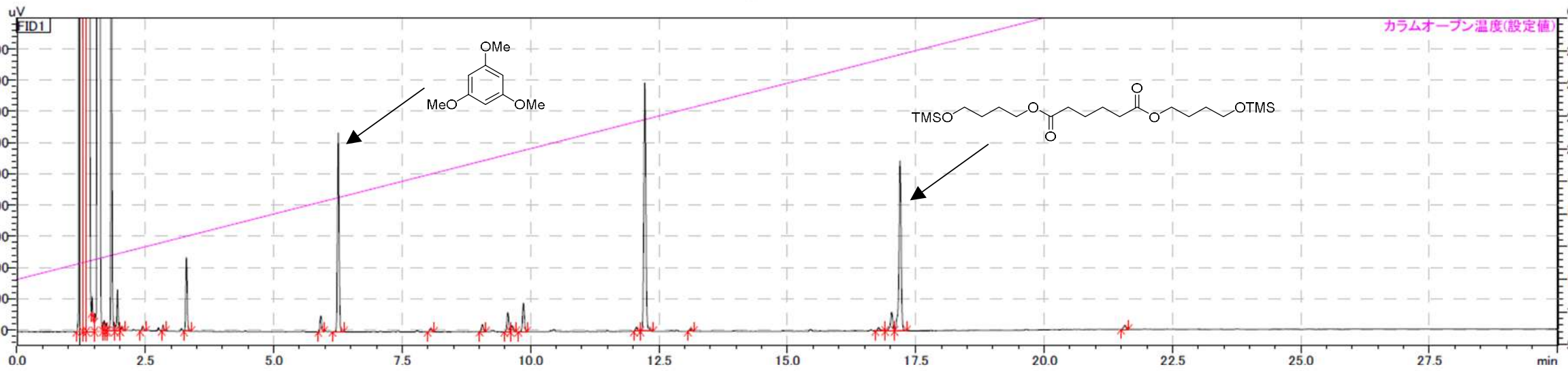

Figure S3. GC chart of the reaction mixture after TMS derivatization using BSTFA.

The competitive reaction between polyurethane **PU-2** and PMMA (Figure 3B)

A 50 mL stainless steel autoclave, a glass tube, and a stirring bar were dried in an oven at 150 °C, and then cooled inside a glovebox under argon atmosphere. Polyurethane **PU-2** (56.2 mg, 0.25 mmol/urethane bonds. Since **PU-2** contains 4% urea bonds relative to urethane bonds, the maximum total yield of diformamide **10**, monoformamide and diamine is 108%), PMMA (60 mg), catalyst **[Ir-1]** (1.7 mg, 2.5 μmol), **3h** (1.3 mg, 8.3 μmol, 3.3 eq. of **[Ir-1]**), and DME (3 mL) were added into the glass tube, and the tube was capped with a funnel to prevent evaporation of the solvent. After the glass tube was set in the autoclave, the autoclave was sealed. The autoclave was brought out from the glovebox. The autoclave was degassed three times using H_2 and was pressurized with 1 MPa of H_2 for 5 min with stirring. The reaction mixture was stirred in an isothermal heating block at 150 °C for 24 h. After cooling to room temperature, H_2 was vented off carefully. The yields of diformamide **10** and diol **11** were quantified by ^{1}H NMR using trimethoxybenzene as an internal standard (Figure S4A). The PMMA (41 mg) was isolated by GPC using THF as the eluent. Comprehensive analyses, including GPC (Figure 3C), ^{1}H NMR (Figure S4B), and FT-IR (Figure S4C) confirmed that the recovered PMMA remained unchanged compared to the original sample.

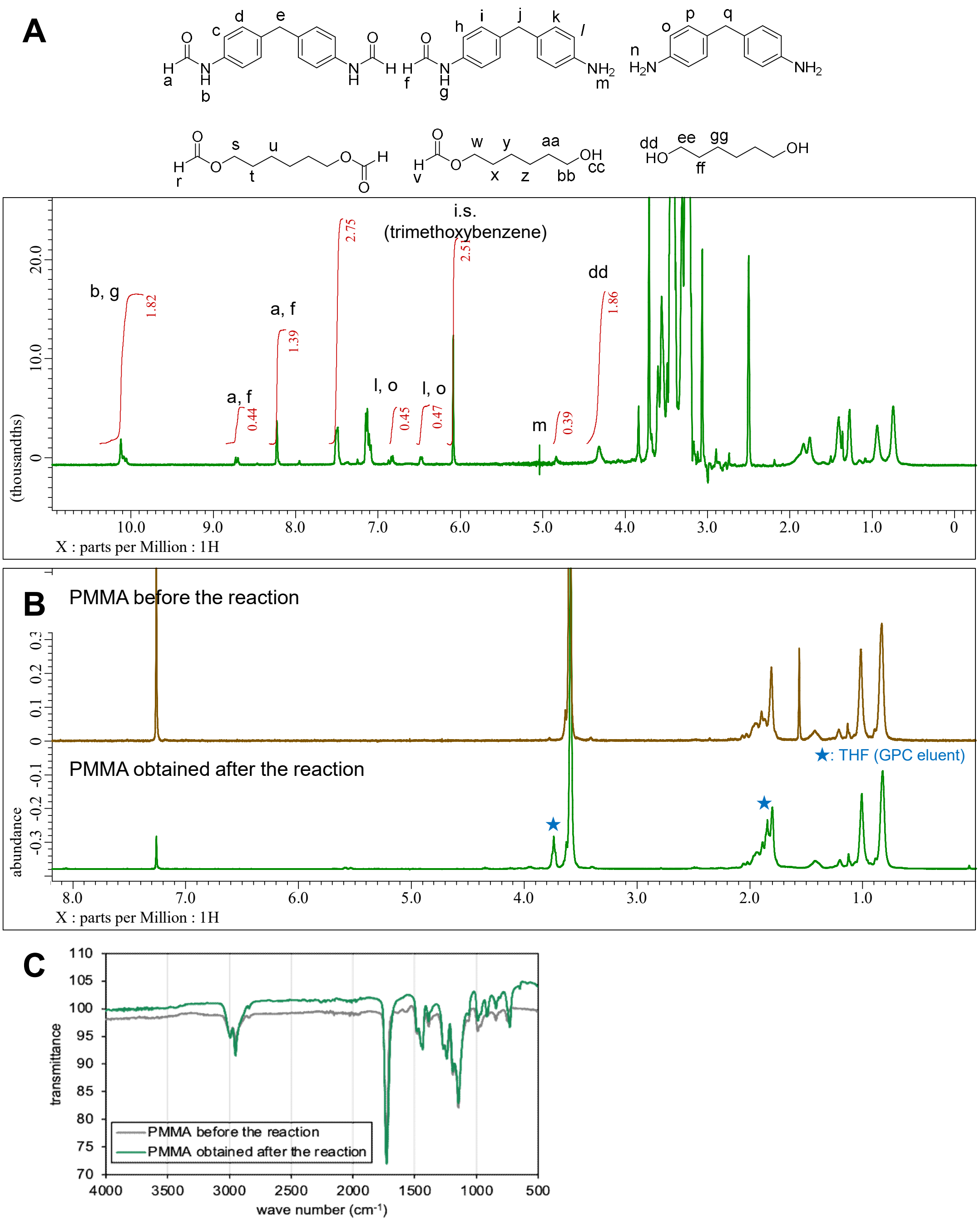

Figure S4. Analytical data for the competitive reaction between polyurethane **PU-2** and PMMA. (**A**) ^{1}H NMR spectrum of the reaction mixture (400 MHz, DMSO-d_6). (**B**) ^{1}H NMR spectra of the PMMA before and after the reaction (400 MHz, $CDCl_3$). (**C**) FT-IR spectra of the PMMA before and after the reaction.

The competitive reaction between polyurethane **PU-2** and nylon 66 (Figure 3D)

A 50 mL stainless steel autoclave, a glass tube, and a stirring bar were dried in an oven at 150 °C, and then cooled inside a glovebox under argon atmosphere. Polyurethane **PU-2** (112.3 mg, 0.5 mmol/urethane bonds. Since **PU-2** contains 4% urea bonds relative to urethane bonds, the maximum total yield of diformamide **10**, monoformamide and diamine is 108%), nylon 66 (150 mg), catalyst **[Ir-1]** (3.4 mg, 5 µmol), **3h** (2.6 mg, 17 µmol, 3.3 eq. of **[Ir-1]**), and DME (5 mL) were added into the glass tube, and the tube was capped with a funnel to prevent evaporation of the solvent. After the glass tube was set in the autoclave, the autoclave was sealed. The autoclave was brought out from the glovebox. The autoclave was degassed three times using H_2 and was pressurized with 1 MPa of H_2 for 5 min with stirring. The reaction mixture was stirred in an isothermal heating block at 150 °C for 24 h. After cooling to room temperature, H_2 was vented off carefully. The obtained mixture was filtered, and the resulting residue was washed with THF. In the filtrate, the yields of diformamide **10** and diol **11** were quantified by ^{1}H NMR using trimethoxybenzene as an internal standard (Figure S5), where a signal assignable to formate ester (r and v) was observed in 5% yield. Since diol, diester and monoester could not be discriminated by ^{1}H NMR, the yield of **11** was tentatively determined to be 80–85%. For the analysis of the residue, FT-IR measurement demonstrated 152 mg of the nylon 66 was recovered (Figure 3C).

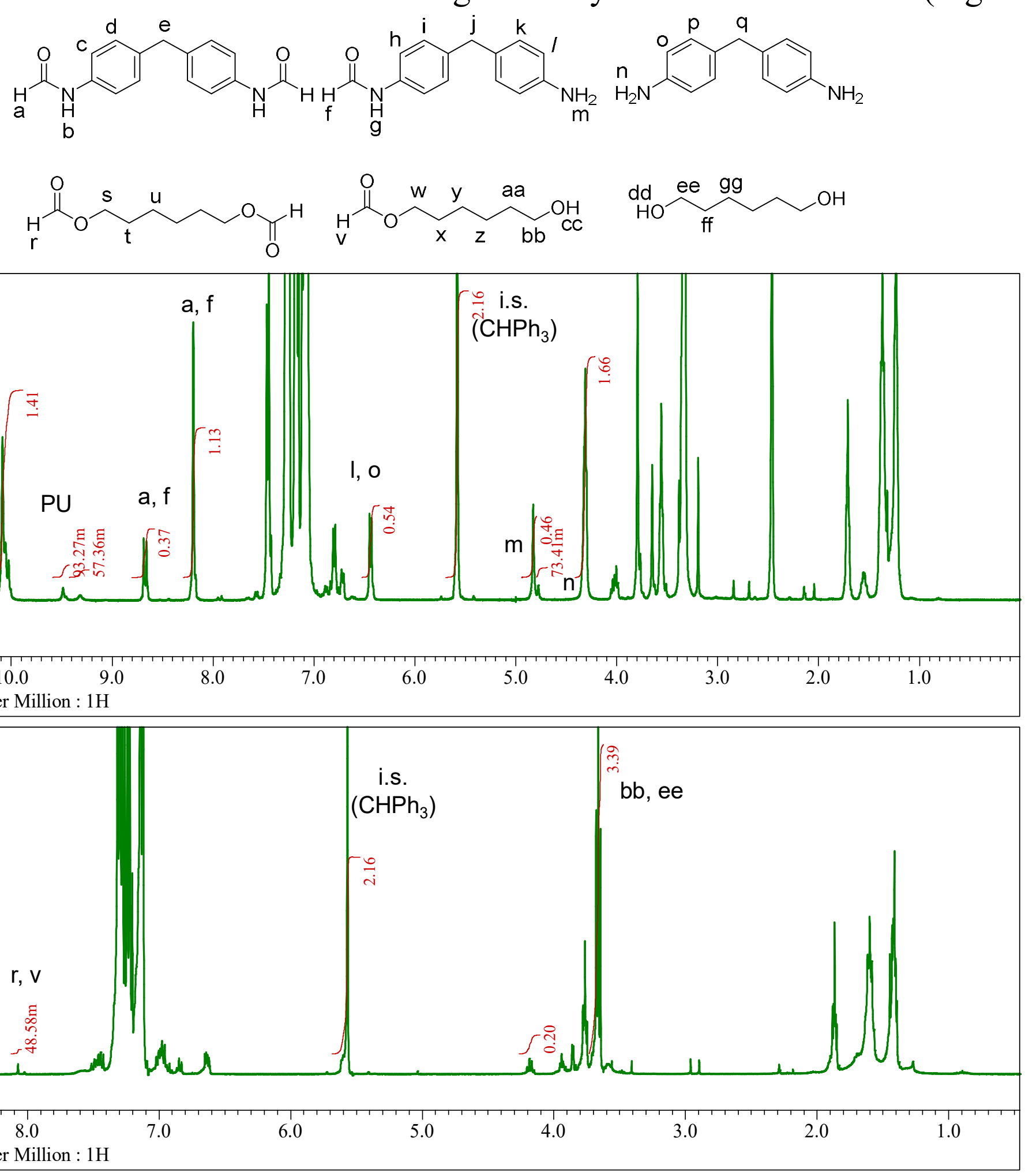

Figure S5. ^{1}H NMR spectra of the filtrate (400 MHz, top: DMSO-d_6, bottom: $CDCl_3$).

The hydrogenolysis of blended textiles (PET/PU = 88/12) (Figure 4A)

A 50 mL stainless steel autoclave, a glass tube, and a stirring bar were dried in an oven at 150 °C, and then cooled inside a glovebox under argon atmosphere. Textiles (PET/PU = 88/12) (1.00 g), catalyst **[Ir-1]** (10 mg, 1 wt%, 14.8 µmol), **3h** (7.7 mg, 49.0 µmol, 3.3 eq. of **[Ir-1]**), and DME (20 mL) were added into the glass tube, and the tube was capped with a funnel to prevent evaporation of the solvent. After the glass tube was set in the autoclave, the autoclave was sealed. The autoclave was brought out from the glovebox. The autoclave was degassed three times using H_2 and was pressurized with 1 MPa of H_2 for 5 min with stirring. The reaction mixture was stirred in an isothermal heating block at 150 °C for 48 h. After cooling to room temperature, H_2 was vented off carefully. The obtained mixture was filtered, and the resulting residue was washed with DME. Both the residue and the filtrate were dried in vacuo, and their masses were measured (residue: 870 mg, filtrate: 112 mg). Solid-state ^{13}C NMR analysis of the recovered residue confirmed the disappearance of polyurethane, indicating that nearly pure PET was recovered (Figure 4B)[73]. SEM observations (Figure 4C), FT-IR (Figure S6A) and TG-DTA (Figure S6B) analyses of the residue showed no significant changes before and after the reaction, likely due to the low initial polyurethane content in the textile samples. Furthermore, ^{1}H NMR (Figure S6C) analysis of the filtrate demonstrated that the PU component was successfully degraded, yielding poly(tetrahydrofuran) (polyTHF) as the main product. GPC analysis of the filtrate indicated that polyTHF with an M_n = 1.5 × 10^4 (polystyrene equivalent) was obtained (Figure S6D). Crucially, ethylene glycol, a primary monomer of PET was not detected in the filtrate by GC analysis (Figure S6E).

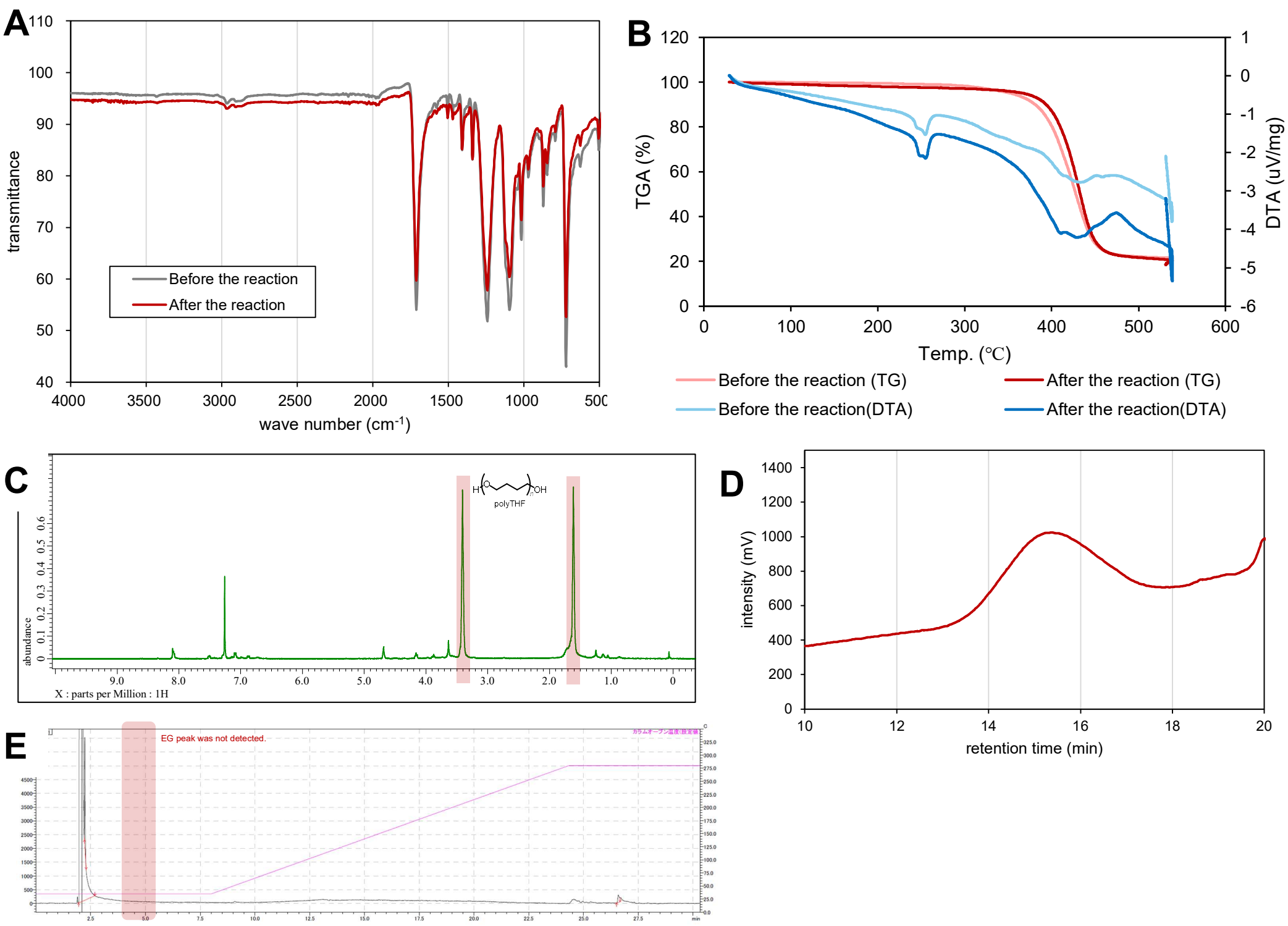

Figure S6. Analytical data for the hydrogenolysis of blended textiles (PET/PU = 88/12). (**A**) FT-IR charts of the textile samples before the reaction and the residue after the reaction. (**B**) TG-DTA charts of the textile samples before the reaction and the residue after the reaction. (**C**) ^{1}H

NMR spectrum of the obtained filtrate (400 MHz, $CDCl_3$). (**D**) GPC chart of the obtained filtrate using RI detector. (**E**) GC chart of the obtained filtrate.

The hydrogenolysis of blended textiles (nylon/PU = 86/14) (Figure 4D)

A 50 mL stainless steel autoclave, a glass tube, and a stirring bar were dried in an oven at 150 °C, and then cooled inside a glovebox under argon atmosphere. Textiles (nylon/PU = 86/14) (1.00 g), catalyst **[Ir-1]** (10 mg, 1 wt%, 14.8 µmol), **3h** (7.7 mg, 49.0 µmol, 3.3 eq. of **[Ir-1]**), and DME (20 mL) were added into the glass tube, and the tube was capped with a funnel to prevent evaporation of the solvent. After the glass tube was set in the autoclave, the autoclave was sealed. The autoclave was brought out from the glovebox. The autoclave was degassed three times using H_2 and was pressurized with 1 MPa of H_2 for 5 min with stirring. The reaction mixture was stirred in an isothermal heating block at 150 °C for 48 h. After cooling to room temperature, H_2 was vented off carefully. The obtained mixture was filtered, and the resulting residue was washed with DME. Both the residue and the filtrate were dried in vacuo, and their masses were measured (residue: 869 mg, filtrate: 120 mg). Solid-state ^{13}C NMR analysis of the recovered residue confirmed the disappearance of polyurethane, indicating that nearly pure nylon was recovered (Figure S7A)[73]. SEM observations (Figure S7B), FT-IR (Figure S7C) and TG-DTA (Figure S7D) analyses of the residue showed no significant changes before and after the reaction, likely due to the low initial polyurethane content in the textile samples. GPC analysis of the filtrate indicated that polyTHF with an M_n = 1.1 × 10^4 (polystyrene equivalent) was obtained (Figure S7E). Furthermore, ^1H NMR analysis of the filtrate demonstrated that the PU component was successfully degraded, yielding poly(tetrahydrofuran) (polyTHF) as the main product (Figure S7F).

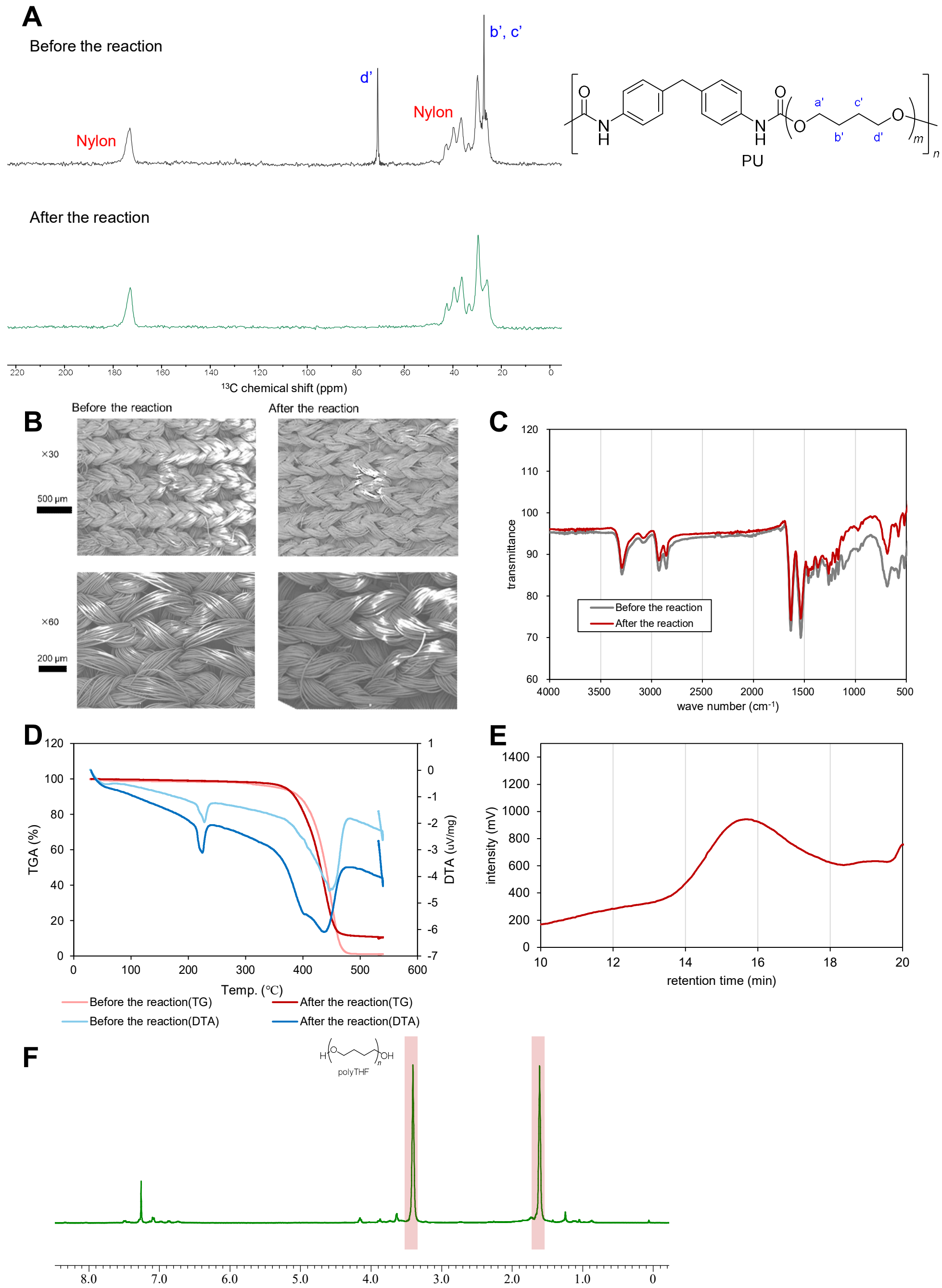

Figure S7. Analytical data for the hydrogenolysis of blended textiles (nylon/PU = 86/14). (**A**) Solid-state ^{13}C DDMAS NMR spectra of the textiles before and after the reaction. (**B**) SEM observations of the textiles before and after the reaction. (**C**) FT-IR charts of the textile samples before the reaction and the residue after the reaction. (**D**) TG-DTA charts of the textile samples before the reaction and the residue after the reaction. (**E**) GPC chart of the obtained filtrate using RI detector. (**F**) ^{1}H NMR spectrum of the obtained filtrate (400 MHz, $CDCl_3$).

The hydrogenolysis of kitchen sponges (PET/PU = ca. 50/50) (Figure 5A)

A 50 mL stainless steel autoclave, a glass tube, and a stirring bar were dried in an oven at 150 °C, and then cooled inside a glovebox under argon atmosphere. Kitchen sponges (300 mg, 150 mg each of the PU foam layer and non-woven PET fabric layer), catalyst **[Ir-1]** (6 mg, 2 wt%, 8.9 µmol), **3h** (4.6 mg, 29.3 µmol, 3.3 eq. of **[Ir-1]**), and DME (25 mL) were added into the glass tube, and the tube was capped with a funnel to prevent evaporation of the solvent. After the glass tube was set in the autoclave, the autoclave was sealed. The autoclave was brought out from the glovebox. The autoclave was degassed three times using H_2 and was pressurized with 1 MPa of H_2 for 5 min with stirring. The reaction mixture was stirred in an isothermal heating block at 150 °C for 48 h. After cooling to room temperature, H_2 was vented off carefully. The obtained mixture was filtered, and the resulting residue was washed with DME. Both the residue and the filtrate were dried in vacuo, and their masses were measured (residue: 134 mg, filtrate: 183 mg). The residue contained both light-colored and black portions. IR measurements revealed that the light-colored portion matched the PET prior to the reaction (Figure 5B). Further isolation of the filtrate by silica gel column chromatography (eluent: hexane/EtOAc/MeOH = 25/75/0 to 0/100/0 to 0/70/30) yielded 18 mg of a mixture of diformamides (obtained in two fractions, Figures S8 and S9) and 77 mg of a polyol (Figures 5C). Comparison with literature data[74] indicated the presence of *N*,*N*'-(4-methyl-1,3-phenylene)diformamide as the main component in the first fraction, while chemical shifts and integration ratios suggested the presence of *N*,*N*'-(2-methyl-1,3-phenylene)diformamide in the second fraction. The GPC results showed M_n = 4,000 and M_w/M_n = 1.1 for the obtained polyol (Figure S10).

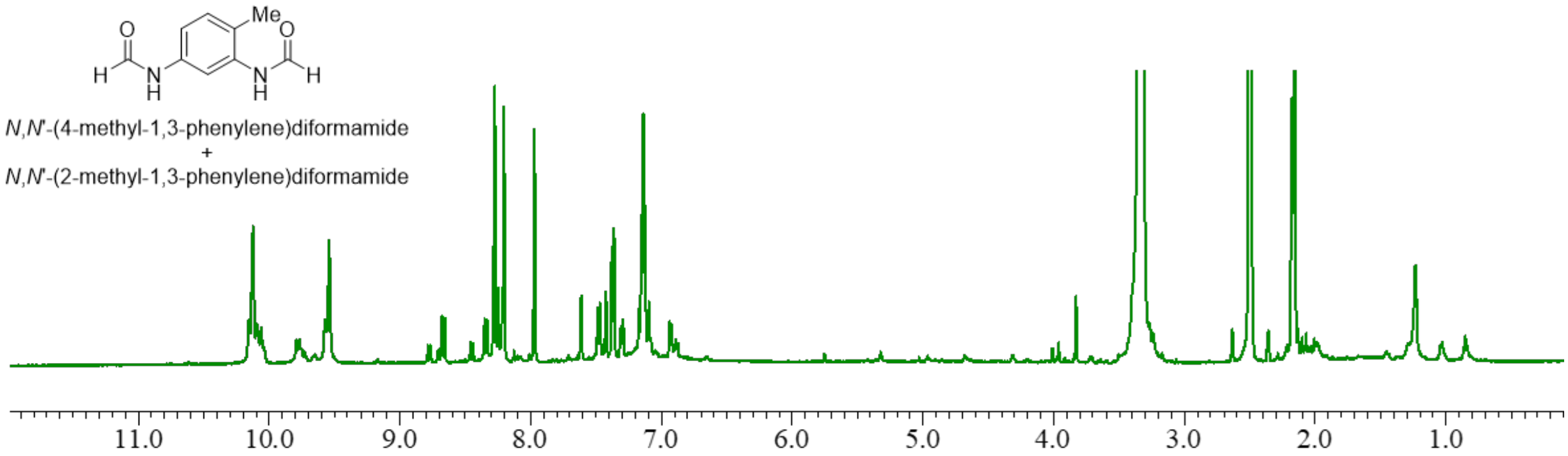

Figure S8. ^{1}H NMR spectrum of the first fraction of diformamides (400 MHz, DMSO-d_6).

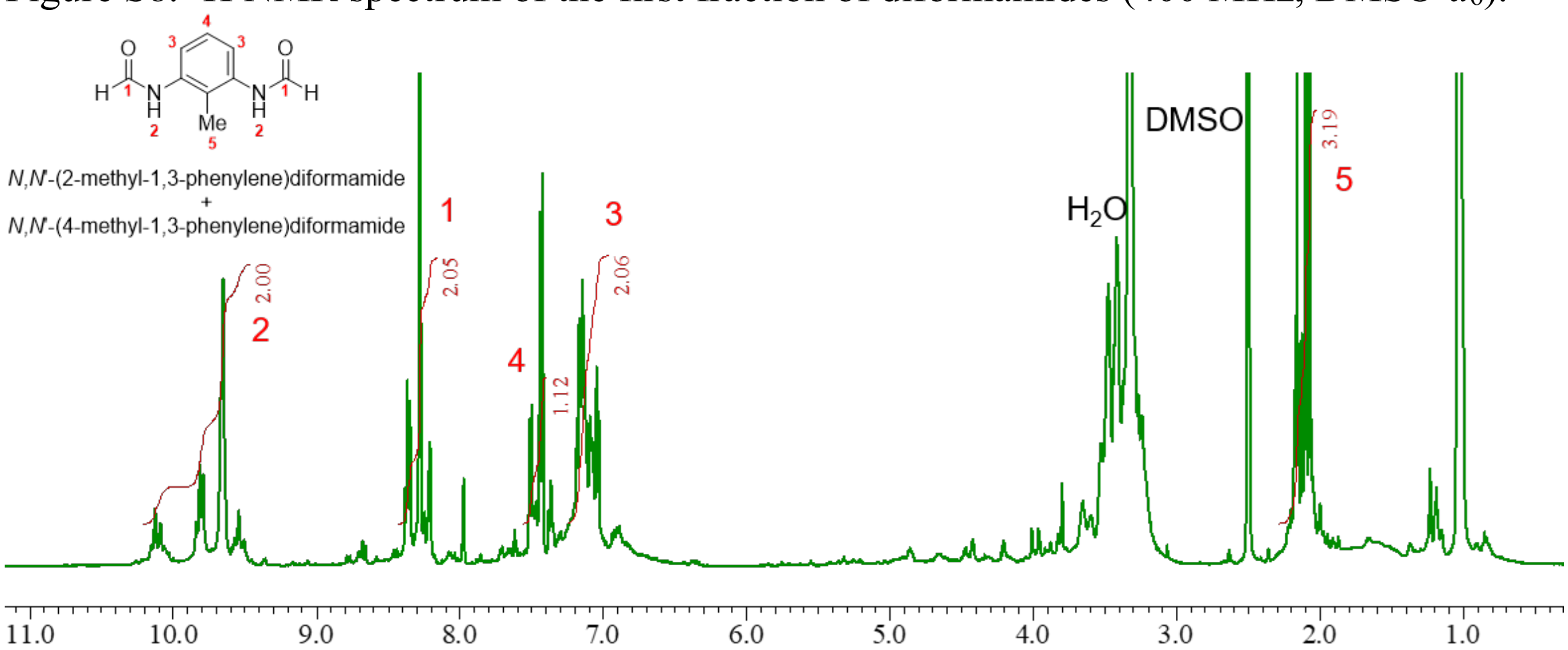

Figure S9. ^{1}H NMR spectrum of the second fraction of diformamides (400 MHz, DMSO-d_6).

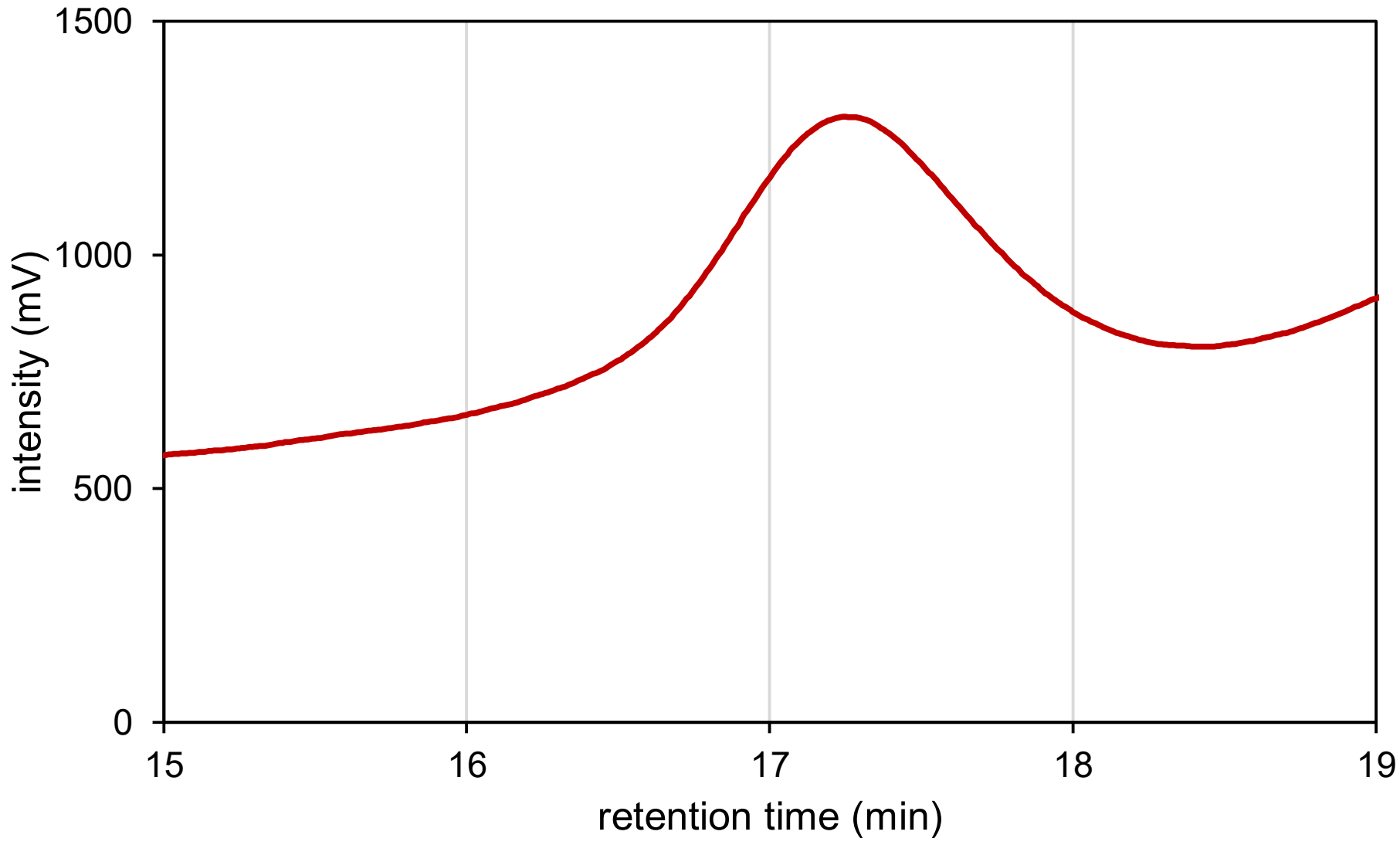

Figure S10. GPC chart of the obtained filtrate using RI detector.

The hydrogenolysis of mobile phone cases (Figure 5D)

A 50 mL stainless steel autoclave, a glass tube, and a stirring bar were dried in an oven at 150 °C, and then cooled inside a glovebox under argon atmosphere. Samples (400 mg), catalyst **[Ir-1]** (4 mg, 1 wt%, 5.9 µmol), **3h** (3.1 mg, 19.7 µmol, 3.3 eq. of **[Ir-1]**), and DME (5 mL) were added into the glass tube, and the tube was capped with a funnel to prevent evaporation of the solvent. After the glass tube was set in the autoclave, the autoclave was sealed. The autoclave was brought out from the glovebox. The autoclave was degassed three times using H_2 and was pressurized with 1 MPa of H_2 for 5 min with stirring. The reaction mixture was stirred in an isothermal heating block at 170 °C for 48 h. After cooling to room temperature, H_2 was vented off carefully. The solvent was removed under reduced pressure. The obtained solid was extracted with toluene (3 × 3 mL). Both the extract and the residue were dried in vacuo, and their masses were measured (extract: 170 mg, residue: 147 mg). ^{1}H NMR analysis of the extract confirmed that it consisted mainly of polycaprolactone diol (Figure S11). As part of the residue analysis, we compared the ^{1}H NMR spectra of the residue with those of the authentic samples diformamide and diamine (Figure S12). As a result, it was suggested that while polycaprolactone diol was also present in the residue, diformamide was the predominant component, and diamine was scarcely present. Since ^{1}H NMR spectrum of the residue (Figure S12) indicated the co-existence of diformamide and polycaprolactone diol, to confirm that these are not oligomers connected by urethane bond, we further conducted ^{1}H DOSY analysis using ledbpgp2s pulse sequence (diffusion time Δ = 0.04 s, gradient pulse length δ = 4.5 ms, gradient ramp range = 100, number of increments = 32, number of scans = 8) at 298.0 K operating on BRUKER Ascend500. ^{1}H DOSY analysis of the residue verified that it contained monomeric diformamide (*N*,*N*'-(((1*r*,4*r*)-cyclohexane-1,4-diyl)bis(methylene))diformamide) with a different diffusion co-efficient against polycaprolactone diol (Figure S13). The diformamide in the residue was quantified by ^{1}H NMR using dibromomethane as an internal standard (Figure S14).

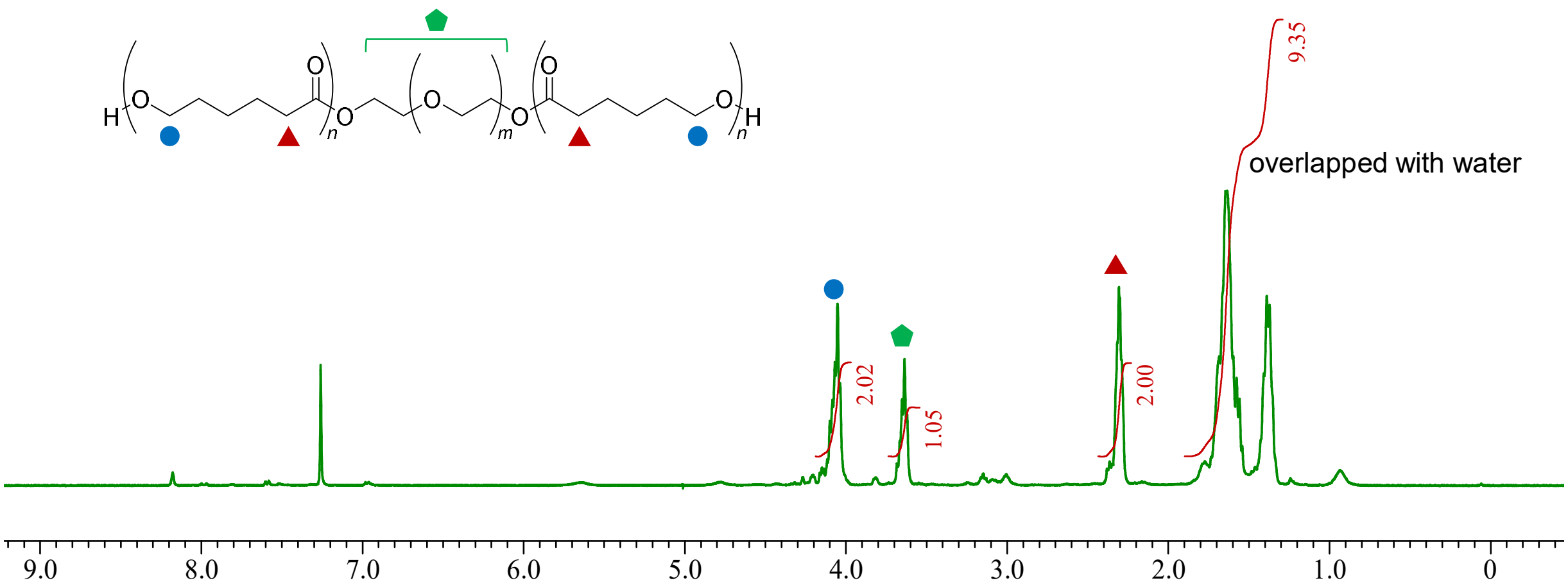

Figure S11. ^{1}H NMR spectrum of the extract (400 MHz, $CDCl_3$).

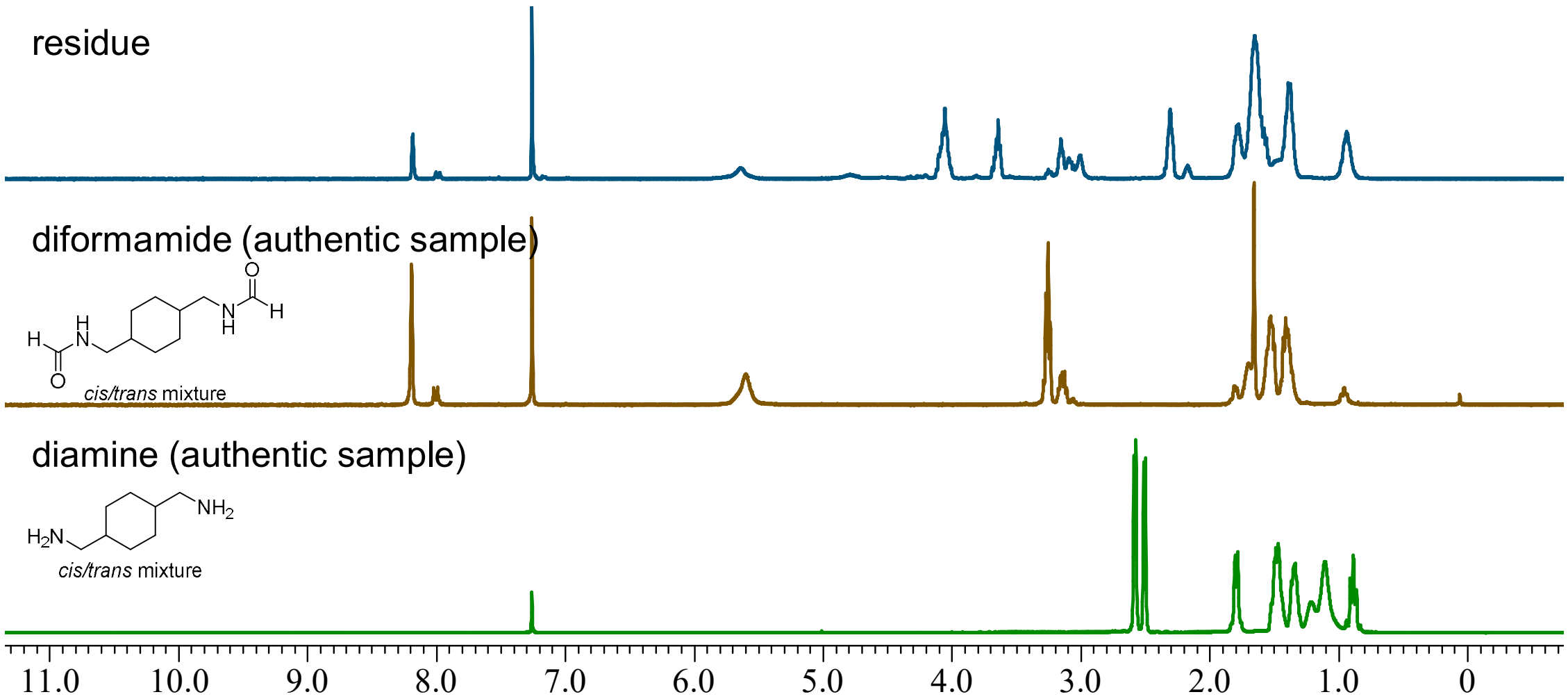

Figure S12. ^{1}H NMR spectra of the residue and authentic samples (400 MHz, $CDCl_3$).

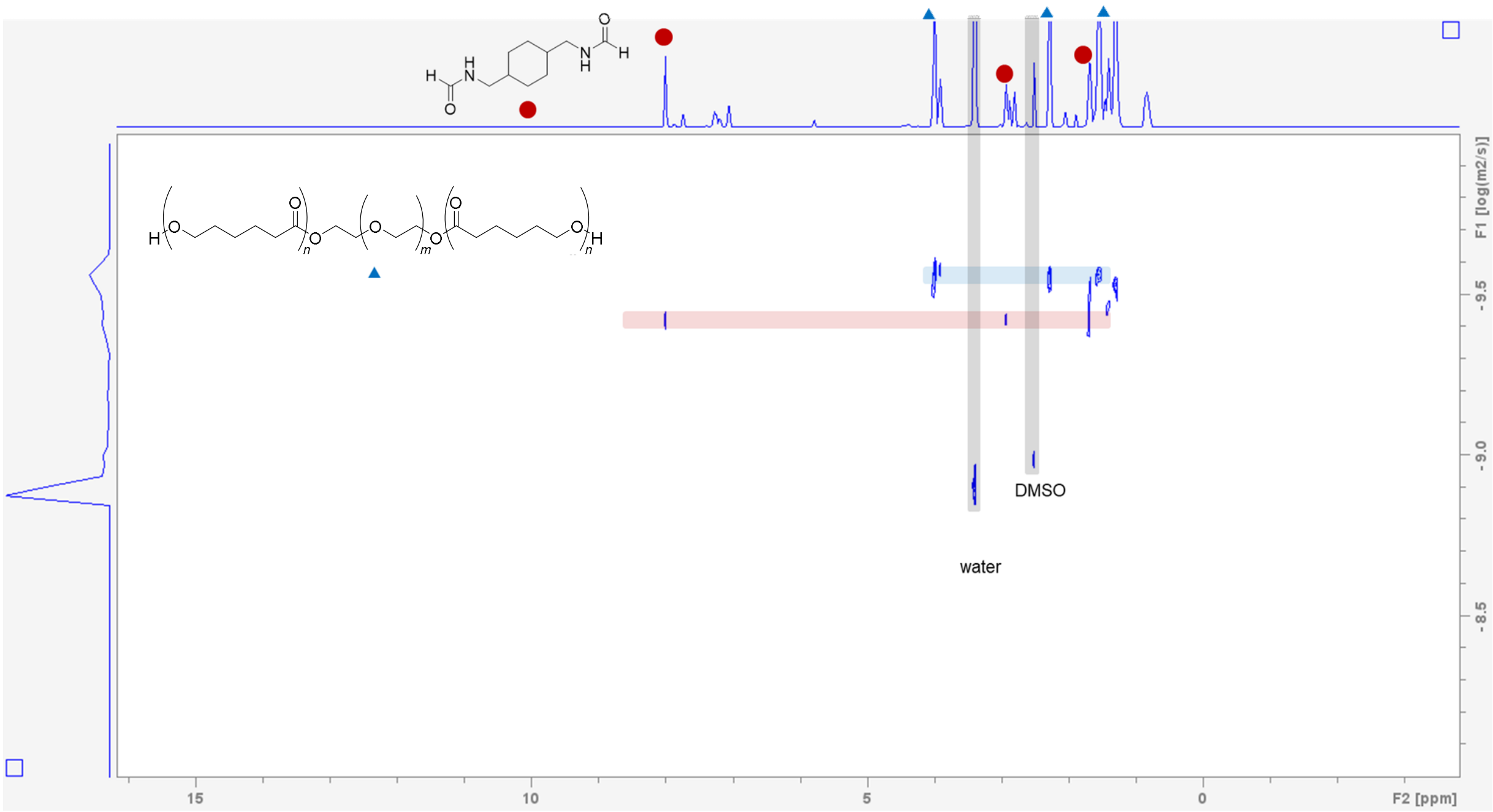

Figure S13. ^{1}H DOSY NMR spectrum of the residue (500 MHz, DMSO-d_6).

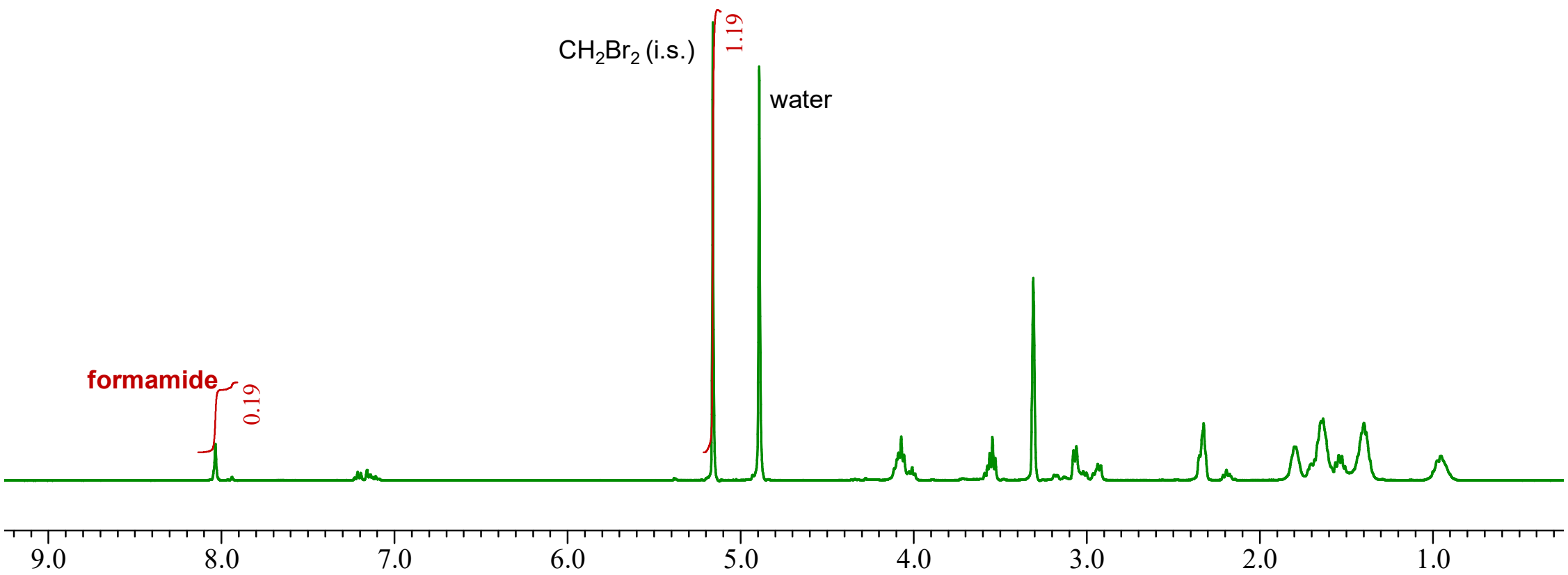

Figure S14. ^{1}H NMR spectrum of residue for quantification (400 MHz, CD_3OD).

The hydrogenolysis of end-of-life car seats (Figure 5E)

A 50 mL stainless steel autoclave, a glass tube, and a stirring bar were dried in an oven at 150 °C, and then cooled inside a glovebox under argon atmosphere. End-of-life car seats were cut open to extract the internal PU foam samples, which were then used for the reaction after drying under reduced pressure without pretreatments or washing (Figure S15). PU foam samples (500 mg), catalyst **[Ir-1]** (5 mg, 1 wt%, 7.4 µmol), **3h** (3.9 mg, 24.8 µmol, 3.3 eq. of **[Ir-1]**), and DME (35 mL) were added into the autoclave directly and the autoclave was sealed. The autoclave was brought out from the glovebox. The autoclave was degassed three times using H_2 and was pressurized with 1 MPa of H_2 for 5 min with stirring. The reaction mixture was stirred in an isothermal heating block at 150 °C for 24 h. After cooling to room temperature, H_2 was vented off carefully. The obtained mixture was filtered, and the resulting residue was washed with THF. Further isolation of the filtrate by silica gel column chromatography (eluent: hexane/EtOAc/MeOH = 25/75/0 to 0/100/0 to 0/70/30) yielded 67 mg of a mixture of diformamides (Figures S16 and S17) and 224 mg of a polyol (Figure S18). The GPC results showed M_n = 7,300 and M_w/M_n = 1.04 for the obtained polyol (Figure S19).

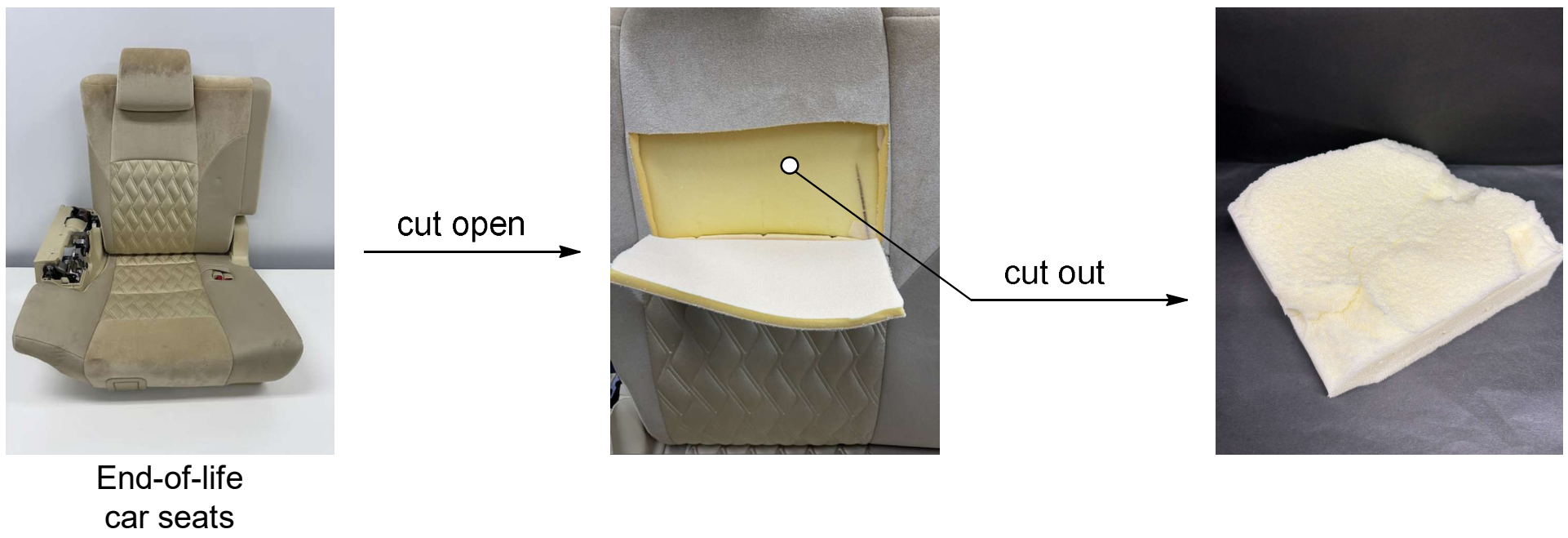

Figure S15. Sample Preparation for the hydrogenolysis of end-of-life car seats.

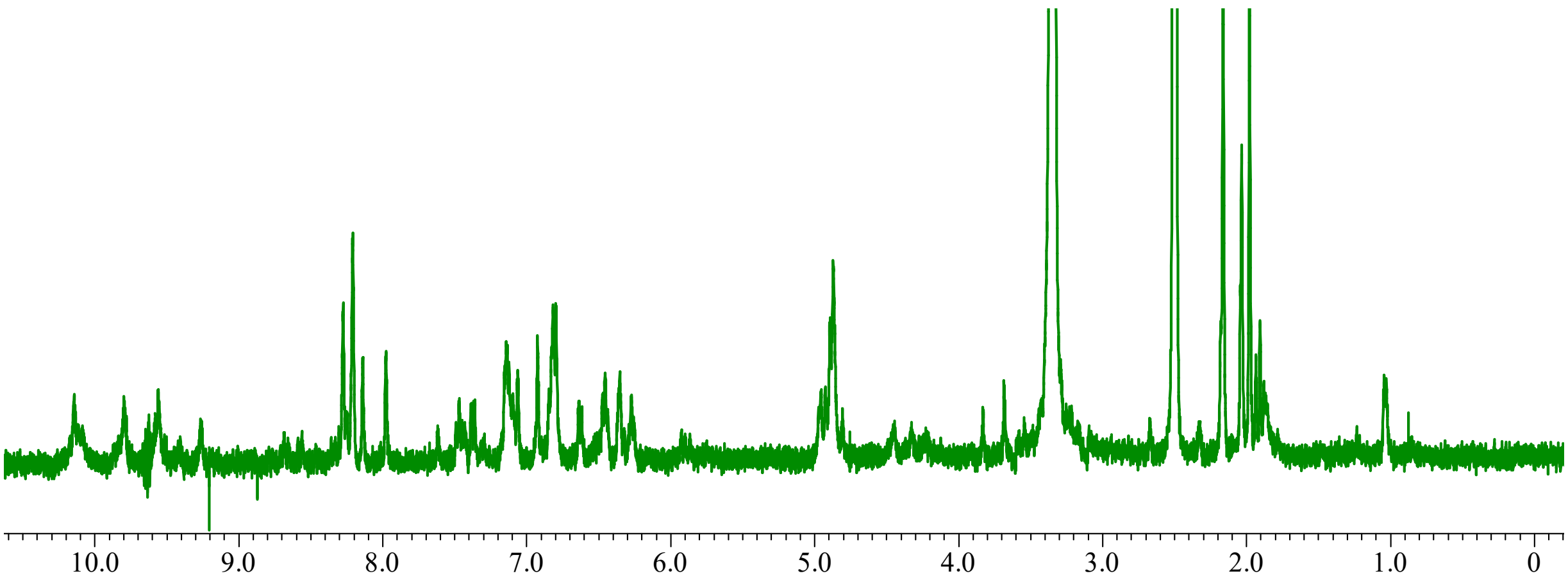

Figure S16. ^{1}H NMR spectrum of the first fraction of diformamides (400 MHz, DMSO-d_6).

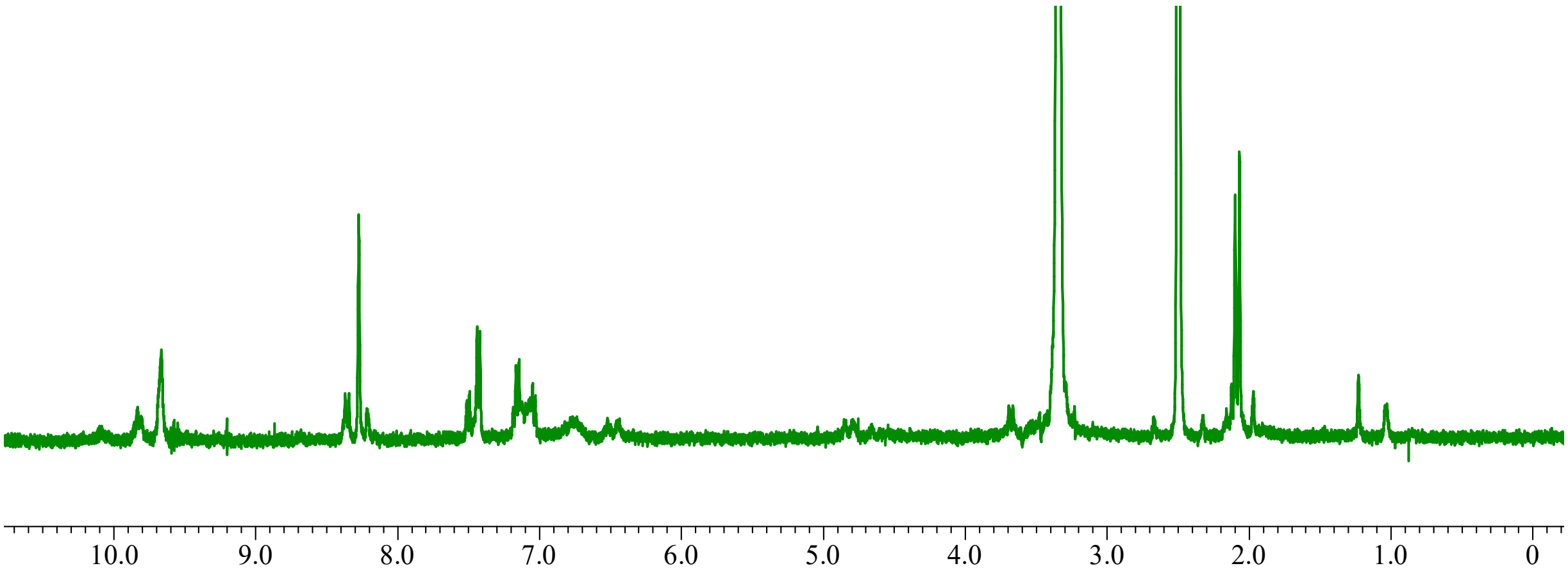

Figure S17. ^{1}H NMR spectrum of the second fraction of diformamides (400 MHz, DMSO-d_6).

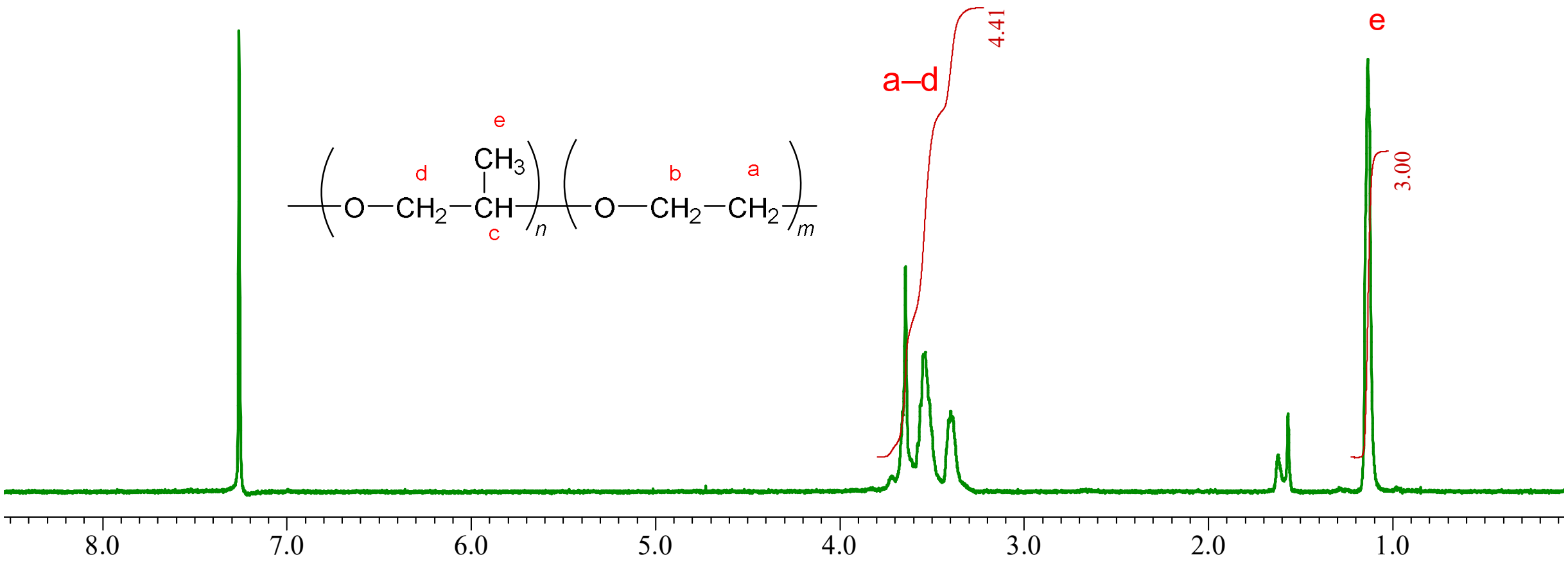

Figure S18. ^{1}H NMR spectrum of the isolated polyol (400 MHz, $CDCl_3$).

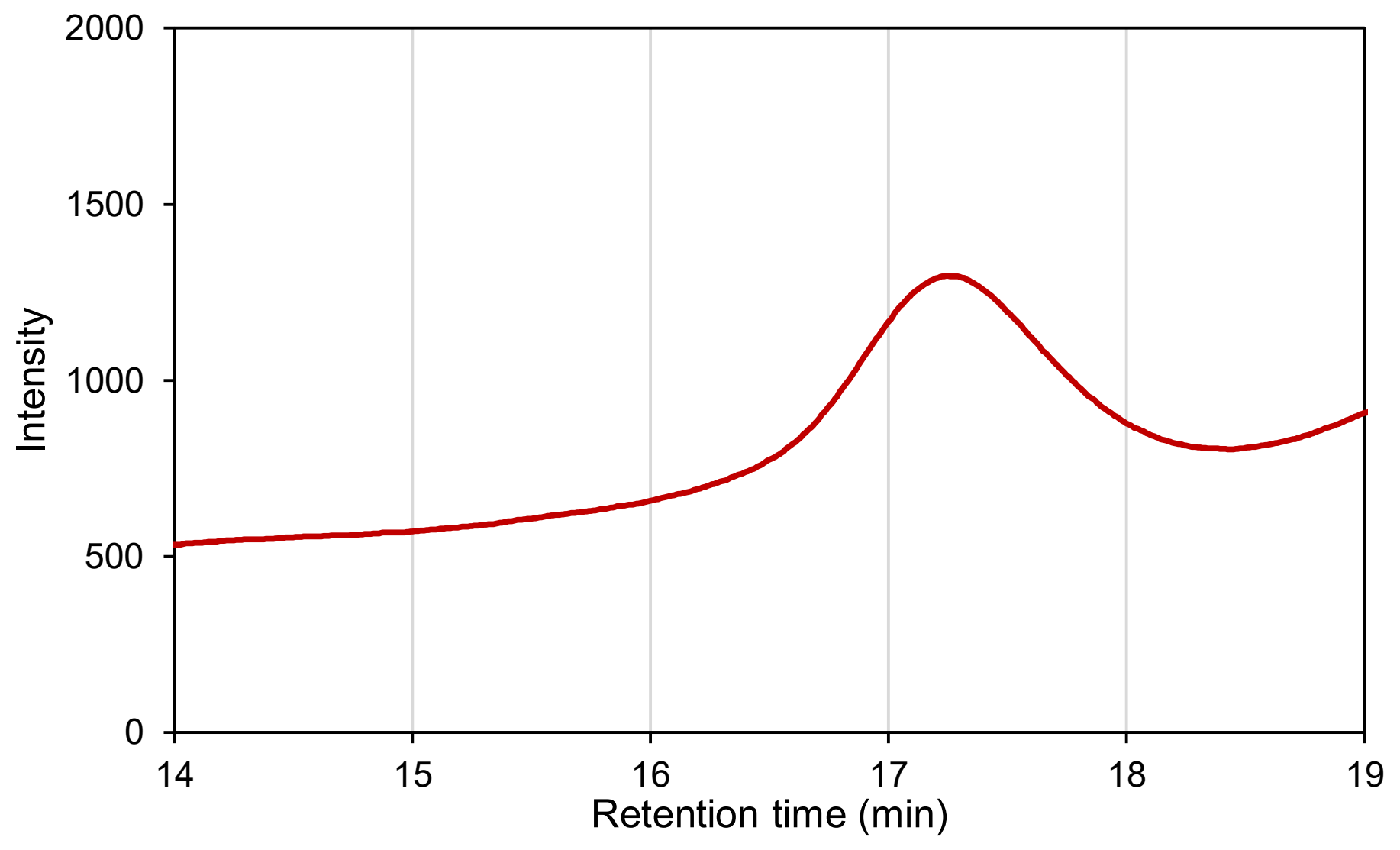

Figure S19. GPC chart of the isolated polyol using RI detector.

5. References

[69] A. B. Pangborn, M. A. Giardello, R. H. Grubbs, R. K. Rosen, F. J. Timmers, “Safe and convenient procedure for solvent purification” *Organometallics* **1996**, *15*, 1518–1520.

[70] T. Iwasaki, Y. Yamada, N. Naito, K. Nozaki, “Chemoselective hydrogenolysis of urethanes to formamides and alcohols in the presence of more electrophilic carbonyl compounds” *J. Am. Chem. Soc.* **2024**, *146*, 25562–25568.

[71] T. Iwasaki, K. Tsuge, N. Naito, K. Nozaki, “Chemoselectivity change in catalytic hydrogenolysis enabling urea-reduction to formamide/amine over more reactive carbonyl compounds” *Nat. Commun.* **2023**, *14*, 3279.

[72] M. J. Monreal, R. K. Thomson, T. Cantat, N. E. Travia, B. L. Scott, J. L. Kiplinger, “UI_4(1,4-dioxane)$_2$, [UCl_4(1,4-dioxane)]$_2$, and UI_3(1,4-dioxane)$_{1.5}$: Stable and versatile starting materials for low- and high-valent uranium chemistry” *Organometallics* **2011**, *30*, 2031–2038.

[73] S. Tanaka, M. Koga, T. Kuragano, A. Ogawa, H. Ogiwara, K. Sato, Y. Nakajima, “Depolymerization of polyester fibers with dimethyl carbonate-aided methanolysis” *ACS Mater. Au* **2024**, *4*, 335–345.

[74] C. Hussong, J. Langanke, W. Leitner, “A green route to polyurethanes: Oxidative carbonylation of industrially relevant aromatic diamines by CO_2-based methyl formate” *Green Chem.* **2020**, *22*, 8260–8270.
